# Identification of the Candidate Proteins Related to Oleic Acid Accumulation during Peanut (*Arachis hypogaea* L*.*) Seed Development through Comparative Proteome Analysis

**DOI:** 10.3390/ijms19041235

**Published:** 2018-04-18

**Authors:** Hao Liu, Haifen Li, Jianzhong Gu, Li Deng, Li Ren, Yanbin Hong, Qing Lu, Xiaoping Chen, Xuanqiang Liang

**Affiliations:** 1 Crops Research Institute, Guangdong Academy of Agricultural Sciences, South China Peanut Sub-Center of National Center of Oilseed Crops Improvement, Guangdong Provincial Key Laboratory of Crop Genetic Improvement, Guangzhou 510640, China; liuhao2054@stu.scau.edu.cn (H.L.); lihaifen@gdaas.cn (H.L.); hongyanbin@gdaas.cn (Y.H.); luqing@gdaas.cn (Q.L.); 2 Peanut Research Institute, Kaifeng Academy of Agricultural Sciences, Kaifeng 475004, China; xinkeyan@126.com (J.G.); dengli_1225@sina.com (L.D.); renli120@sina.com (L.R.)

**Keywords:** peanut, proteome, oleic acid, *FAB2*, fatty acid pathway

## Abstract

Peanuts (*Arachis hypogaea* L.) are an important oilseed crop, containing high contents of protein and fatty acids (FA). The major components of FA found in peanut oil are unsaturated FAs, including oleic acid (OA, C18:1) and linoleic acid (LOA, C18:2). Moreover, the high content of OA in peanut oil is beneficial for human health and long-term storage due to its antioxidant activity. However, the dynamic changes in proteomics related to OA accumulation during seed development still remain largely unexplored. In the present study, a comparative proteome analysis based on iTRAQ (isobaric Tags for Relative and Absolute Quantification) was performed to identify the critical candidate factors involved in OA formation. A total of 389 differentially expressed proteins (DEPs) were identified between high-oleate cultivar Kainong176 and low-oleate cultivar Kainong70. Among these DEPs, 201 and 188 proteins were upregulated and downregulated, respectively. In addition, these DEPs were categorized into biosynthesis pathways of unsaturated FAs at the early stage during the high-oleic peanut seed development, and several DEPs involved in lipid oxidation pathway were found at the stage of seed maturation. Meanwhile, 28 DEPs were sporadically distributed in distinct stages of seed formation, and their molecular functions were directly correlated to FA biosynthesis and degradation. Fortunately, the expression of *FAB2* (stearoyl-acyl carrier protein desaturase), the rate-limiting enzyme in the upstream biosynthesis process of OA, was significantly increased in the early stage and then decreased in the late stage of seed development in the high-oleate cultivar Kainong176. Furthermore, real-time PCR verified the expression pattern of *FAB2* at the mRNA level, which was consistent with its protein abundance. However, opposite results were found for the low-oleate cultivar Kainong70. Overall, the comparative proteome analysis provided valuable insight into the molecular dynamics of OA accumulation during peanut seed development.

## 1. Introduction

*Arachis hypogaea* L*.*, an important branch of the legume species, is commonly known as the peanut or groundnut. Peanuts provide essential nourishment for poverty-stricken populations in less-developed regions throughout tropical areas. Due to its nutritional properties, taste and health-promoting effects, the peanut ranks as having the second highest production among all grains and legumes, with a global yield of more than 40 million tons [1]. Currently, cultivated peanuts are going through an evolution event due to the hybridization of two diploid ancestor wild species, *Arachis duranensis* (AA type) and *Arachis ipaenis* (BB type), resulting in induction of spontaneous chromosome doubling and formation of an allopolyploid (AABB type genome, 2n = 4x = 40) [2]. Comparative karyotyping analysis illustrates that chromosome organization is highly conserved in the peanut and its two putative progenitors, particularly in the B genome chromosomes. However, recent comparative karyotyping has shown that multiple hybridization and chromosome integration events of *A. ipaensis* with diverse varieties of *A. duranensis* have probably occurred in the peanut derived from the two diploid ancestors [3]. At the present stage, the entire genomes of *A. duranensis* and *A. ipaensis* have been sequenced and analyzed, laying the first step in understanding the evolution of the tetraploid genomes in peanuts as well as facilitating studies on the genetics, breeding and gene function in peanuts [4,5].

The production of peanuts has rapidly increased over previous years, and it has become an important cash crop for smallholders, thus alleviating rural poverty. In 2014, more than 43.9 million tons of groundnuts with shells were harvested from nearly 26.5 million ha [6]. Moreover, the peanut seed contains approximately 45–56% oil and 24% protein, as well as fatty acids (FA) and an excellent stability due to the presence of natural antioxidants. Peanut oil contains a high level of unsaturated FAs (USFAs) (more than 80%), of which around 50% is oleic acid (OA). Diets with high levels of OA have been shown to have positive effects on human health, including lowering cholesterol levels, decreasing the risk of coronary heart diseases, and preventing hypertension [1]. Because of its dietary and health benefits, the market demand for peanut oil is continuously growing. In addition, the prospective breeding objective is to exploit application-directed studies associated with breeding practices to breed market-oriented new peanut varieties with high OA [7]. Even though a great deal of attention has been paid to high-oleate peanut breeding programs, the detailed molecular mechanisms of OA formation still remain largely unclear. In terms of chemical structure, OA has one double bond at the δ9 position of the hydrocarbon chain, and this feature guarantees OA to be more stable compared with linoleic acid (LOA) [8]. Currently, *Arabidopsis FAD2* homologous genes *AhFAD2A* and *AhFAD2B* have been identified from the peanut A and B sub-genomes, respectively. Both of them encode the δ12 FA desaturase (*FAD2*) and dominantly control the critical step in transformation from OA to LOA, while a recessive mutant allele at the *FAD2* locus, *fad2*, exhibits defects in this transformation, causing the high-oleate content in peanuts [9]. However, systemic elucidation of OA formation has not been carried out in peanuts in previous studies, and other genes associated with OA synthesis should be further explored.

Proteome-based high-throughput analyses generate huge datasets related to biological processes of peanut development. Comparative analyses have revealed fundamental molecular mechanisms and complex lipid networks as well as lipid dynamics in the peanut seed [10]. In addition, gravitropic response proteins involved in differentially operated pathways in the peanut gynophore have been explored by utilizing the proteomic analysis, and comparative analyses of proteomics and transcriptomes have also identified potential candidate proteins for future functional studies on early swelling pods of peanuts [11,12]. However, based on the experimental results of comparative proteomics, no evidence supports the mechanism of OA biosynthesis in high-oleate peanut cultivars. Therefore, the integration of proteomic approaches will greatly accelerate the characterization of high OA formation during the seed development of peanuts.

In order to further understand the mechanisms underlying the high OA formation in peanuts, we utilized an iTRAQ (isobaric Tags for Relative and Absolute Quantification)-based method to investigate the differentially expressed proteins (DEPs) related to the FA biosynthesis pathway at six seed developmental stages in high-oleate cultivar Kainong176 and low-oleate cultivar Kainong70. The comparative proteome analysis was able to sufficiently assess the high OA formation by multiple candidate proteins that contributed to FA biosynthesis and degradation. Our findings provide valuable insight into the molecular dynamics of OA accumulation during peanut seed development.

## 2. Results

### 2.1. Morphology and Oil Accumulation of Developing Peanut Seeds

To explore the detailed fluctuations in the FA profile during seed formation, we documented the phenotype characteristics of seed development in the peanut. Initially, the gynophore elongated into the soil and then rapidly expanded its volume to form the pod that was loaded with white, spongy tissues. Secondly, the size of the seeds constantly increased, along with the pods rapidly swelling; then, the spongy tissues gently decreased. Finally, seeds grew more and more slowly until becoming mature, and the original thick, spongy tissue was transformed into a thin, white layer that clung to the inner surface of the shell. The peanut shell became dehydrated and transformed into a hard outer shell.

**Figure 1:**
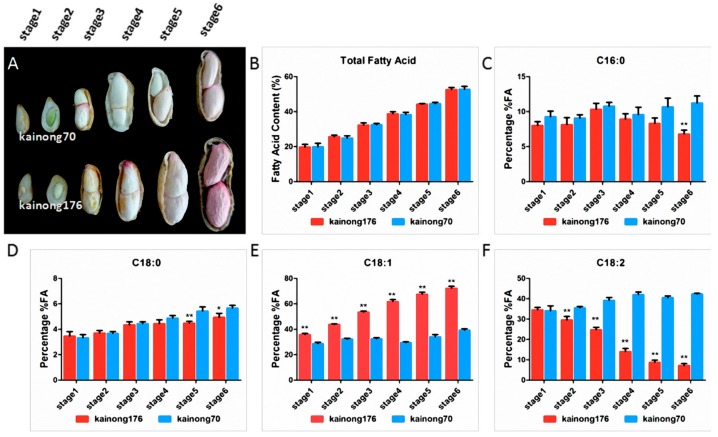
Morphological and dynamic changes of fatty acids (FAs) during peanut seed development. (**A**) Seed samples were collected from different development stages in high-oleate cultivar Kainong176 and normal-oleate cultivar Kainong70. Stages 1–6 represent the six stages from 20 days-after-flowering (DAF) to 70 DAF. (**B**–**F**) These histograms display the dynamic changes in total FA, palmitic acid (C16:0), stearic acid (C18:0), oleic acid (OA) (C18:1) and linoleic acid (LOA) (C18:2), respectively. Values are show as the means (±SD) of three biological replicates, and asterisks indicate a significant difference (* *p* < 0.05, ** *p* < 0.01) compared with the normal-oleate cultivar, Kainong70.

Furthermore, to determine the variation in FA composition in peanut seeds, the total contents of FA, OA, and LOA were examined from the two varieties at six stages that encompassed notable time-nodes in development (Figure 1A). The oil content of the cotyledon in both varieties began to obviously form as early as stage 2, and a fast increase appeared again until the seeds reached the maturity stage (Figure 1B). No difference in total FA and stearic acid (C18:0) contents were observed between the two cultivars, but the palmitic acid (C16:0) content in low-oleate cultivar Kainong70 was lower compared with Kainong176 at the maturity stage (Figure 1C,D). OA and LOA were the highest components of FA. Moreover, the total content of OA was consistently accumulated during seed development in the high-oleate cultivar, while the content of LOA gradually decreased. In contrast, Kainong70 contained a significantly lower OA content than Kainong176, and the relative proportions of OA and LOA in low-oleate cultivar Kainong70 were stable during seed development (Figure 1E,F).

### 2.2. Mass Spectrometry Analysis and Protein Identification

The iTRAQ method was utilized to identify the DEPs between two peanut cultivars at six developmental stages. Two independent biological repeats were simultaneously performed. More than 200,000 unique mass spectra were generated for each replicate (Figure S1A). After searching against the peanut transcription database, a total of 86, 119 and 91,901 unique peptides were obtained from Kainong70 and Kainong176, respectively (Figure S1B). Finally, a total of 7201 and 7666 proteins were successfully identified in the peanut cultivars, respectively (Figure S1C). Experimental data were normalized, and a principal component analysis and clustering analysis showed that the repeatability and accuracy of the obtained proteome data were relatively high (Figure S2). In addition, a heat map representing the expression levels of 2310 DEPs was generated (Figure S3).

### 2.3. Identification of DEPs (Differentially Expressed Proteins) during Seed Development in Low-Oleate Cultivar, Kainong70

**Figure 2:**
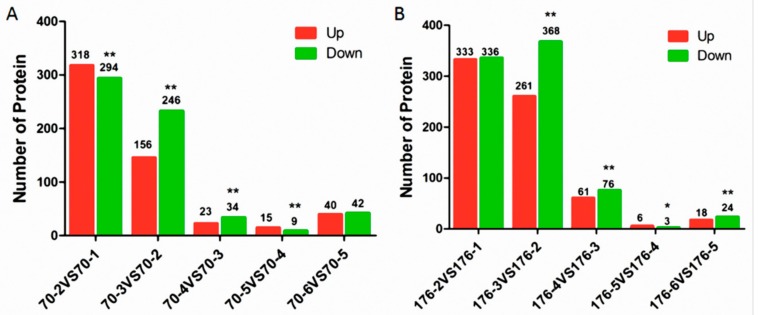
Statistical analysis of differentially expressed proteins (DEPs) in high-oleate cultivar, Kainong176, and low-oleate cultivar, Kainong70. (**A**) Statistical analysis of DEPs at different seed development stages in low-oleate cultivar, Kainong70; (**B**) Statistical analysis of DEPs at different seed development stages in high-oleate cultivar, Kainong176. The asterisks indicate a significant difference (* *p* < 0.05, ** *p* < 0.01) between the number of downregulated DEPs compared with the number of upregulated DEPs.

A total of 552 upregulated and 625 downregulated DEPs were identified at six stages in low-oleate cultivar, Kainong70 (Figure 2A and Table S1). The DEPs were mainly concentrated in the early stages of seed development, from stage 1 to stage 3, accounting for nearly 85.9% and 86.4% of total upregulated and downregulated DEPs, respectively. To understand the functions of DEPs, all quantified proteins were blasted against the Uniprot-GOA (Gene Ontology Annotation) database. Based on the GO (Gene Ontology) analysis, the DEPs were categorized into 20 biological processes, 23 cellular compartments and 20 molecular functions. In terms of biological processes, small molecule metabolic process and single-organism metabolic process were the two major groups in the early stage. The catabolic process, response to abiotic stimulus and organophosphate metabolic process played decisive roles in later stages. Cytoplasm, intracellular organelle parts and intracellular organelles were the top three cellular compartments. Catalytic activity, oxidoreductase activity, binding, copper ion binding, mRNA binding, protein binding and transition metal binding were the major molecular functional groups in different stages (Figure S4). Furthermore, KEGG (Kyoto encyclopedia of genes and genomes) pathway categorization based on the functional DEPs indicated that the most highly-enriched biological pathways in the peanut seeds were metabolic pathways, biosynthesis of secondary metabolism, and carbon metabolism (Figure S6). However, the KEGG analysis showed that 15 DEPs could be classified as being involved in FA metabolism, 11 DEPs were involved in FA biosynthesis, and seven DEPs were involved in FA degradation in the early development stages (stage 1 to stage 3). Approximately 49 identified DEPs were involved in FA metabolism, and these DEPs were categorized into three groups based on their biological processes, as follows: FA biosynthetic process, lipid oxidation and FA metabolism process (Table 1).

### 2.4. Identification of DEPs during Seed Development in High-Oleate Cultivar Kainong176

In addition, a total 679 upregulated and 807 downregulated proteins were identified at six stages in the high-oleate cultivar, Kainong176 (Figure 2B and Table S2). A total of 1486 DEPs were successfully mapped into 20 different Clusters of Orthologous Groups (COG) categories, and single-organism metabolic processes, small molecule metabolic processes, responses to stress, protein binding and purine-containing compound metabolic processes were the most frequently detected categories at different seed developmental stages. Generally, the GO results in the high-oleate cultivar, Kainong176, based on cellular components and molecular functions, were similar to those of Kainong70 (Figure S5). The KEGG pathway enrichment analysis indicated that metabolic pathways, biosynthesis of secondary metabolism and carbon metabolism were the three primarily-enriched pathways (Figure S7). The KEGG pathway classification revealed that the number of DEPs in Kainong176 categorized into the FA pathway was almost identical to that of the low-oleate cultivar, Kainong70, while six DEPs were specifically mapped to the unsaturated FA biosynthesis pathway at stage 3 in Kainong176, suggesting that this stage might serve as a critical time for OA formation or accumulation in the high-oleate peanut variety (Table 2).

### 2.5. Identification of DEPs at the Same Developmental Stage in Different Cultivars

**Figure 3:**
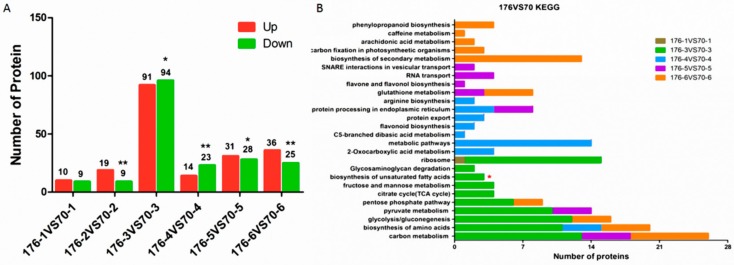
The DEPs identified at different seed developmental stages in Kainong176 and Kainong70. (**A**) Statistical analysis of DEPs at different seed developmental stages in Kainong176 and Kainong70. The asterisks indicate a significant difference (* *p* < 0.05, ** *p* < 0.01) between the number of downregulated DEPs compared with number of the upregulated DEPs. (**B**) KEGG pathway enrichment analysis of DEPs at different seed developmental stages in Kainong176 and Kainong70.

To investigate the different expressions between distinct peanut cultivars at the same developmental stages, DEPs were compared between the protein datasets of Kainong176 and Kainong70 by applying a cutoff p-value of <0.05. The DEP distribution analysis displayed that there were 19, 28, 185, 37, 59 and 61 DEPs from stage 1 to stage 6, respectively (Figure 3A and Table S3). The number of DEPs obtained in stage 3 was greater than that of other developmental stages, indicating the involvement of complex developmental events during stage 3, particularly the biological process of transformation from stearoyl (C18:0) into OA (C18:1). The functions of the identified DEPs were classified using GO assignment. A total of 389 proteins were divided into 27 functional GO items. Most cellular component assignments were tied to the cytoplasm (Figure S8). A pathway analysis was used to categorize function annotations for all annotated proteins based on the KEGG database. We found that 389 DEPs could be categorized into 26 pathways—metabolic pathways were dominant, and most DEPs were tightly related to carbohydrate metabolism. The biosynthesis of unsaturated FAs specifically occurred in stage 3, while the DEPs identified from stage 2 were not enriched in any pathway (Figure 3B). In the annotation of FA and lipid signaling pathways, seven DEPs were annotated to FA biosynthetic and metabolic pathways, 16 DEPs were annotated to lipid signaling pathways, and six DEPs were functionally correlated with lipid synthesis (Table 3). Additionally, 28 DEPs related to FA biosynthesis and degradation were found to be distributed between the six stages of seed formation in Kainong176 and Kainong70 (Table 3). Next, we investigated the protein–protein interaction network of 28 DEPs in STRING (https://string-db.org) using their homologous genes from Arabidopsis. These results indicated that the complicated network contained 24 nodes, and the crucial nodes included Araip.10039818.1 (AT1G24360), Araip.10037384.1 (EMB3003), Araip.10037915.1 (LTA2), Araip.10005278.1 (AT2G34590), Araip.10021150.1 (EFE) (Figure S9). Interestingly, FAB2, encoding a stearoyl-ACP desaturase (SACPD) [13] was involved in stearoyl desaturation to produce OA, and the expression of this protein was first increased at stage 3 and then decreased at stage 6. The analysis of these pathways provided valuable information on the potential key factors involved in OA formation.

### 2.6. Analysis of Expressions of DEPs at the mRNA Level

**Figure 4:**
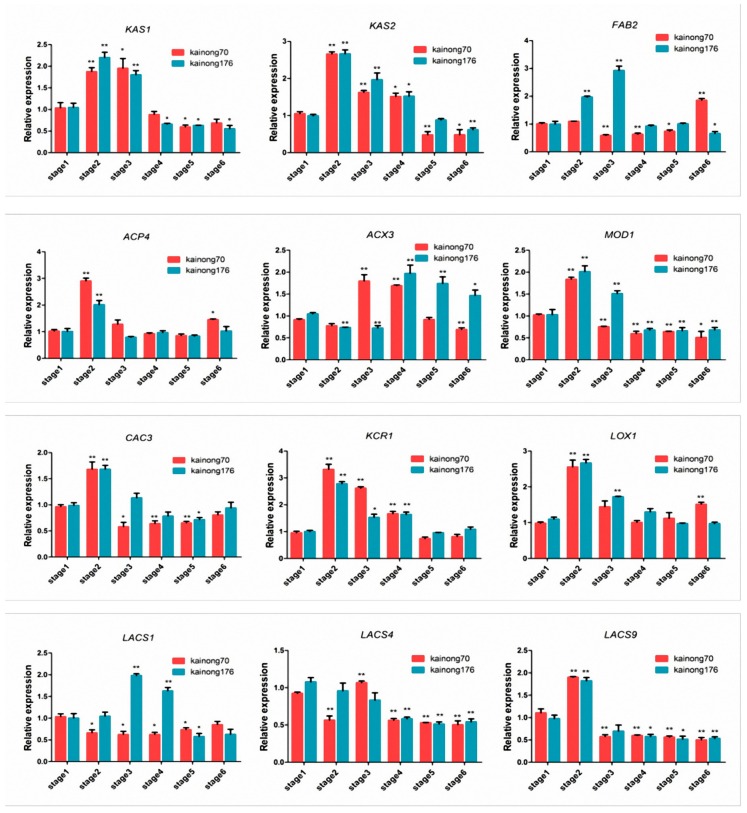
Relative transcription levels of genes involved in FA biosynthesis and degradation at each stage of seed development in Kainong176 and Kainong70. Each measurement was carried out in triplicate with three biological replicates, and the values are expressed as means ± SE (* *p* < 0.05, ** *p* < 0.01) compared with the low-oleate cultivar, Kainong70.

To validate the ITRAQ results and determine whether the differences in protein abundances corresponded to their transcriptional levels, quantitative real-time PCR was performed to examine the expressions of genes associated with FA and lipid accumulation and degradation at six continuous stages between Kainong176 and Kainong70. *Ah18S* and stage 1 samples were designated as the endogenous control and the reference samples, respectively. According to the proteomic results, 12 genes were subsequently characterized to confirm their expression at the mRNA level. Transcriptional analyses showed that the expression of 12 genes significantly changed during seed development (Figure 4). The relative expression patterns of *KAS1* [14], *KAS2* [15], *LOX1* [16], *LACS9* [17], *KCR1* [18] and *ACP4* [19] at the mRNA level were consistent with their levels of protein expression at different stages. However, the expression of *CAC3* [20], *ACX3* [21] *MOD1* [22], *LACS1* [23] and *LACS4* [24] at the mRNA level was not completely consistent with the proteomic results. Moreover, the expression of *FAB2* [25] was first decreased in the early stages and then increased in the late stage of seed development (stage 6) in Kainong70. In contrast, its expression in the high-oleate variety, Kainong176, was significantly increased in the early stages (stage 1–3) and then continuously reduced at the stage of seed maturation. This result suggests that *FAB2* plays a pivotal role in the biological processes of OA formation. Furthermore, we found that 16 mutant sites existed in the high-oleic variety, Kainong176, compared with the normal-oleic variety, Kainong70, after cloning of the coding sequence of *FAB2* (Figure S11A–C). The *FAB2-GFP* fusion protein was located at the chloroplasts in the protoplast cells of *Arabidopsis* (Figure S11D).

## 3. Discussion

In the present study, we performed a comprehensive proteome analysis to identify the expression profile of proteins involved in the OA synthesis pathway during seed development in high- and normal-oleic peanut varieties. A total of 28 DEPs were identified to participate in the regulation of FA biosynthesis and degradation (Table S3), such as *KASI*, *KASII*, *FAB2*, acetyl-CoA family proteins and *LCAS* family proteins. Our study provides valuable information for improving the quality of peanut oil.

In plants, the production of C16:0 and C18:0 FA is mainly performed by multiple monofunctional enzymes, such as FA synthase (FAS). The malonyl-thioester reacts with acetyl-CoA and acyl-ACP acceptors to form a carbon–carbon bond as the initial substrate of FA synthesis, which is the first restricted step, controlled by the condensing enzymes, 3-ketoacyl-ACP synthases (*KAS*). The initial condensation reaction of acetyl-CoA and malonyl-ACP is catalyzed by *KAS* isoform III (*KASIII*) [26], and subsequent 4-carbon production (3-ketobutyrl-ACP) requires *KASI* to form C16:0-ACP, whereas the final elongation of the 16-carbon palmitoyl–ACP to the 18-carbon stearoyl–ACP is catalyzed by a third condensing enzyme, *KASII* [27]. According to the proteomic analysis, the expressions of *KASI* and *KASII* are concurrently and significantly increased at stage 2 and then decreased at stage 6 in both peanut cultivars, and this common characteristic of *KAS* expression pattern implied that *KAS* enzymes mainly contribute to the biosynthesis of palmitoyl–ACP (C16:0-ACP) and stearoyl–ACP (C18:0-ACP), while OA (C18:1) accumulation was found to coincide with seed development [28].

**Figure 5:**
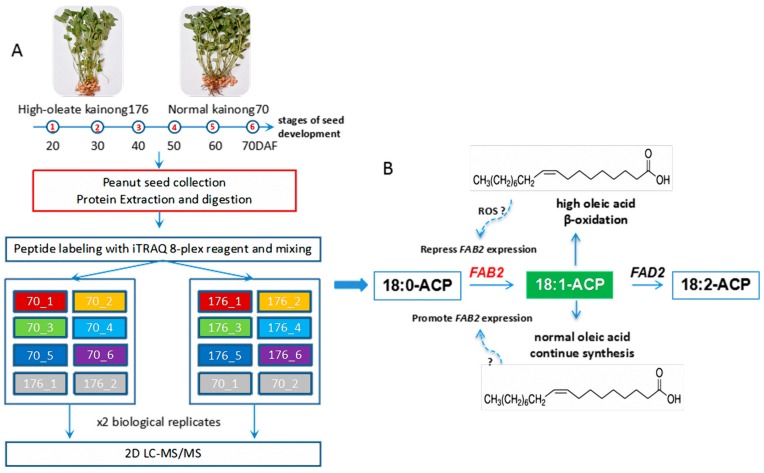
Model of OA feedback which regulates the expression of *FAB2* in the high-oleate cultivar. (**A**) Brief procedure of proteomic data obtained by iTRAQ; (**B**) Putative model of OA feedback which regulates *FAB2* at both the mRNA and protein levels. The question-marks (?) indicate some kinds of unidentified regulatory pathways.

Alternatively, *SACPD* is a key rate-limiting enzyme that catalyzes the conversion of stearic acid (18:0) to OA (18:1) during de novo FA biosynthesis and produces monounsaturated FAs in plant cells [29]. Currently, there are seven *SACPD* isoforms in the *Arabidopsis thaliana* genome, and *FAB2* (*SSI2*), encoding ∆9 stearoyl–ACP desaturases (*SADs*) efficiently desaturate C18:0 to form *cis*-Δ-9 C18:1 (OA), whereas the molecular details of other members in the *SACPD* family remain poorly understood [30,31]. In this study, we found that the *FAB2* expression at the mRNA level exhibited a similar trend to its protein abundance, which was a significant increase in the late stage of seed development in the low-oleate cultivar, Kainong70. On the contrary, the abundance of *FAB2* proteins dramatically decreased at stage 3 and reached its lowest level at stage 6 in Kainong176 (Figure 4). Accompanied by the fluctuation in *FAB2* expression, whereby levels were increased and then decreased, OA was quickly accumulated in the early stages, but we speculate that there was an unknown regulatory mechanism continuously suppressing *FAB2* expression during seed development (Figure 5). The comparative proteome analysis indicated that OA was accumulated to a certain threshold, leading to activation of certain biological reactions related to OA synthesis. Therefore, the expression of *FAB2* was downregulated in the high-oleate cultivar, Kainong176. Actually, maturing seeds accumulate compounds that are remobilized to establish post-germinative seedlings, and the majority of FAs found in embryonic oil of peanut are OA and LOA. If the relative proportion of OA was much less than the normal level in total reserved compounds, the signaling of shortage could be easily examined by the interior receptor, and the biosynthetic systems would be activated via transiently synthesizing a mass of *FAB2* to promote OA accumulation, therefore leading to elevated expression of *FAB2* at the terminal stage of seed maturation in the low-oleate variety, Kainong70 (Figure 5B). Accordingly, a previous study cloned the *AhFAB2* gene from the peanut genome, but the molecular function of *AhFAB2* remains elusive in the groundnut [32]. Here, the *FAB2* coding sequence exhibited genetic diversity in both peanut varieties (Figure S11A–C), and the protein was located at chloroplast (Figure S11D), implying that *FAB2*-controlled stearic acid (C18:1-ACP) was exported from chloroplasts into the endoplasmic reticulum to promote OA synthesis through the acetyl-coenzyme A transport pathway. Combined with our data, the overexpression of *AhFAB2* in peanut cultivar could be used as a potential strategy to improve the OA content in peanut oil. Meanwhile, the results of *FAB2* suggest that the iTRAQ-identified proteins provide a genetic resource for investigating the mechanisms of OA or FA synthesis in future studies.

Furthermore, the transformation from OA to LOA is mainly catalyzed by *Δ12 FAD2*, which also catalyzes the conversion of monounsaturated FAs to polyunsaturated FAs [33]. In addition, genetic studies have demonstrated that the high content of OA in groundnuts is controlled by two homozygous recessive mutant genes, *AhFAD2A* and *AhFAD2B*. Due to the characteristics of *AhFAD2*, conventional approaches have been widely applied in breeding practices, and products conform to the market demand of peanut oil with a high content of OA [34]. Nonetheless, the significant expression of *AhFAD2* was not shared at any period of seed filling in the DEP profiles for both peanut varieties. This result suggests that the enzyme activity of *AhFAD2* was maintained at a relatively constant level for oleic formation over the entire period of seed development, which is different from previous data that indicated that the expression of *AhFAD2* is first increased in the early stages and then decreased at the late stage [10].

Plant *de novo* FA synthesis occurs in the plastid. Since no transport of acetyl-coenzyme A (CoA) between sub-cellular compartments can be demonstrated in plant cells, plastidial acetyl-CoA is probably the unique building block used for FA production, and the major straightforward mechanism of the acetyl-CoA pool requires the plastidial pyruvate dehydrogenase complex (PDC) to rapidly generate acetyl-CoA [35,36]. Meanwhile, a large number of proteins associated with FA biosynthesis pathway were observed during peanut seed development in the current study (Table 3). *E1-Beta-2* (pyruvate dehydrogenase E1β subunit) [37] and *LTA2* (dihydrolipoyllysine-residue acetyltransferase component 4 of PDC) [38] protein abundances were obviously increased in the high-oleate variety, and pyruvate metabolism was the fourth most enriched pathway identified in the KEGG analysis. We propose that the pyruvate metabolism was beneficial for OA accumulation. Moreover, a full set of enzymes involved in FA β-oxidation have been discovered in the high-oleate strain, including *ACO3* (1-aminocyclopropane 1-carboxylate oxidase 3), *ACX3* (acyl-co enzyme A oxidase 3), enoyl-CoA hydratase 2 (*Araip.10019122*) and 3-oxoacyl reductase (*Araip.10039818*) (Table 3). However, the molecular connection between FA β-oxidation and OA formation is still unclear. We hypothesized that each FA was shortened by a two-carbon fragment as acetyl-coenzyme A, and the products continued to enter into the cycle of OA synthesis. In conclusion, we successfully identified a series of DEPs during peanut seed development, and future study will focus on the molecular functions of proteins involved in OA formation in groundnuts.

## 4. Methods and Materials

### 4.1. Plant Materials

Two peanut cultivars, Kainong176 and Kainong70, with different OA contents in their mature seeds (Kainong176 > 70, Kainong70 < 40) were used in the present study. These two varieties were cultivated to flower at a low position on the stem under normal growth conditions in a field located in the Baiyun District of Guangzhou City (Guangzhou, Guangdong Province, China), and then the withered flowers fell off the curved gynophore due to geotropism. The aerial pegs were firstly marked with a pink tag after flowering, and secondary labeling was conducted at the time that the elongated gynophore initially touched the ground. The development stages of the marked gynophores were precisely recorded in terms of days after flowering (DAF) in high- and normal-oleic varieties. We abandoned many non-elongated first-marked gynophores. When the gynophores entered the soil and developed into pods, we collected the seed at different phases (from 20 to 70 DAF). To reduce error, we tried to choose the pods with similar phenotypes, at the same development stage, when obtaining peanut seeds for total protein extraction. On the other hand, results from several pre-experiments indicated that the proteomics comparison between the species was able to correlate changes in oleate content at distinct developmental stages—because the plant materials were either high-oleic or normal-oleic cultivars, their oleate content displayed obvious differences from seed formation (20 DAF) to maturity (70 DAF) (Figure S10). The peanut seeds were collected at six developmental stages as follows: 20 (stage 1), 30 (stage 2), 40 (stage 3), 50 (stage 4), 60 (stage 5) and 70 (stage 6) DAF (days after flowering). The harvested seeds were quickly frozen in liquid nitrogen, and then their total FA, protein and RNA were extracted for relevant experiments.

### 4.2. Profile Analysis of FAs

Oil content and composition was analyzed using 5 g of seed collected from each developmental stage. The fresh seeds of different stages were ground into a fine powder in liquid nitrogen. The total oil content was calculated following the Soxhlet extraction method, using *n*-hexane [39]. The profile analysis of FAs in peanut seeds was performed by gas chromatography of the methyl ester of FA, in accordance with the National Standard of the People’s Republic of China (GB/T 17377-2008). Briefly, 100 mg of peanut seed was frozen and ground into a fine powder in liquid N2. The obtained powder was mixed with 1.5 mL of chloroform/methanol (2:1, *v*/*v*) and 100 μL of internal standard solution C17:0 (1 mg/mL). The mixture was extracted by ultrasound-assisted extraction for 30 min and centrifuged at 12,000 rpm for 6 min, and then 1 mL of supernatant was transferred into a fresh tube. The FA extracting solution was mixed with 0.2 mL KCl (75%, *w*/*v*) solution and centrifuged at 12,000 rpm for 6 min, and then 400 μL of the substratum chloroform extract phase was transferred into a new glass tube and prepared for methanol esterification. The extracting solution was mixed with 2 mL of sulfuric acid methanol (5:95, *v*/*v*) and incubated in a water bath at 85 °C for 1.5 h. Subsequently, 1 mL of H_2_O and 1 mL of hexane were added into the tube, the mixture was centrifuged at 5000 rpm for 5 min, and 500 μL of the superstratum hexane extract phase was used for gas chromatography (Cat#YLSB076). The individual FA contents were reported as the relative percentages of OA and LOA in the extracted oil.

### 4.3. Protein Extraction and iTRAQ Labeling of Tryptic Peptides

Protein extraction was carried out according to a previously described method [40]. Briefly, about 0.5 g of peanut seed was ground into a fine powder in liquid nitrogen and washed with ice-cold TCA (Trichloroacetic Acid)/acetone until the mixture became clear, and then the mixture was washed with ice-cold acetone twice to remove the residual TCA. After air-drying, the resultant powder was extracted with lysis buffer (7 M urea, 50 mM Tris-HCl and 5 mM Halt™ Protease Inhibitor Cocktail (Thermo Fisher Scientific, Rockford, IL, USA, Cat.# 78430), pH 8.0), and the proteins were precipitated by adding five volumes of cold methanol containing 0.1 M of ammonium acetate. The pellets were dissolved in 8 M of urea containing 10 mM DTT (dl-Dithiothreitol), before being reduced at 56 °C for 1 h and alkylated using 55 mM iodoacetamide (IAM) at room temperature for 45 min in the dark. The protein concentration was quantified with a Bio-Rad protein assay kit (Bio-Rad, Cambridge, MA, USA). Subsequently, 100 µg of protein from each sample was digested with trypsin at 37 °C for 16 h. After trypsin digestion, peptides were desalted with a Strata X C18 column (Phenomenex, Torrance, CA, USA). Peptides were reconstituted in 0.5 M TEAB (Tetraethylammonium Bromide) and processed using 8-plex iTRAQ reagent (SCIEX, Framingham, MA, USA), in accordance with the manufacturer’s instructions. Briefly, one unit of iTRAQ reagent was thawed and reconstituted in 24 μL of isopropanol. The peptides were labeled with the isobaric tags and incubated at room temperature for 2 h. The labeled peptide mixtures were then pooled and dried using a vacuum centrifuge.

### 4.4. LC-ESI-MS Analysis Based on Triple TOF 5600

The mixed iTRAQ-labeled peptides were dissolved in buffer A (5% ACN (Acetonitrile), 95% H_2_O, pH adjusted to 9.8 with ammonia) to 2 mL and loaded onto a HPLC (High Performance Liquid Chromatographyhigh) RP (Reverse Phase) column (250 × 4.6 mm, 5 μm, 100 Å aperture, Gemini C18 column containing 5-μm particles, Phenomenex, Torrance, CA, USA) in a Prominence HPLC system (Shimadzu LC-20AB HPLC Pump system, Kyoto, Japan). The peptides were eluted at a flow rate of 1 mL/min with a gradient of 5% buffer B (5% H_2_O, 95% ACN, pH adjusted to 9.8 with ammonia) for 10 min, 5–35% buffer B for 40 min, and 35–95% of buffer B for 1 min. The elution was monitored by measuring the absorbance at 214 nm, and peptides were collected in one tube/min during the elution period. The collected fractions were then lyophilized, dissolved in 0.1% formic acid, further pooled into 20 fractions to average the protein contents and vacuum-dried. Each fraction was re-suspended in 50 μL of buffer A (2% ACN, 0.1% FA) and centrifuged at 20,000× *g* for 10 min, and the supernatant was measured at 214 nm again, resulting a final peptide concentration of around 0.5 µg/µL. The supernatant was loaded on an LC-20AD nanoHPLC (Shimadzu, Kyoto, Japan) by the auto-sampler and separated on a C18 column (75 μm × 15 cm, 3.6 μm particles, 120 Å aperture) at 300 nL/min with a gradient of 5–30% of solvent B (1% FA, 95% ACN) for 38 min and 30–80% solvent B for 4 min and maintained at 80% for 8 min, continued with a 41 min gradient running at 300 nL/min from 8 to 35% solvent B in 35 min, which rose by 60% in 5 min, and then was maintained at 80% solvent B for 5 min, and finally returned to 5% in 0.1 min and equilibrated for 10 min. Data acquisition was performed with a Triple TOF 5600 System (SCIEX, Framingham, MA, USA) equipped with a Nano-spray III source (SCIEX, Framingham, MA, USA), a pulled quartz tip as the emitter (New Objectives, Woburn, MA, USA), and controlled with software Analyst 1.6 (SCIEX, Framingham, MA, USA). Data were acquired with the following mass spectroscopy conditions: an ion spray voltage of 2.3 kV, a curtain gas of 30 psi, a nebulizer gas of 15 psi, and an interface heater temperature of 150 °C. A high sensitivity mode was used for the whole data acquisition. The resolution was about 30,000. For IDA (Information Dependent Acquisition), survey scans were acquired in 250 ms and as many as 30 product ion scans were collected if a threshold of 120 counts per second (counts/s) was exceeded and with a 2+ to 5+ charge-state. The total cycle time was fixed to 3.3 s. The Q2 transmission window was 100 Da for 100%. Four-time bins were summed for each scan at a pulse frequency value of 11 kHz through monitoring of the 40 GHz multichannel TDC (Time-to-Digital-Converter) detector with four-anode channel detection. An iTRAQ adjust rolling collision energy was applied to all precursor ions for collision-induced dissociation. Dynamic exclusion was set as 1/2 of peak width (15 s), and then the precursor was refreshed off the exclusion list [41].

### 4.5. Database Search

The raw data were combined and converted into excel format using Protein Pilot, version 4.5 (AB, SCIEX). Peptides and proteins were identified using the Mascot search engine (version 2.3.02, Matrix Science, London, UK). Protein sequence data used for the MS/MS search were obtained from UniprotKB (www.uniprot.org). Database searches were limited to tryptic peptides, and iTRAQ 8-plex was selected as a fixed modification, with two missing cleavages allowed and a 95% confidence level. The FDR (False Discovery Rate) filter was applied at the peptide level, and 1% FDR was used to filter the protein identifications. For quantifications, all quantified peptides in one protein were combined to calculate the p-values. The upregulated and downregulated DEPs were identified by applying cutoff Log^2^ (Fold Change) values of >1 and <−1, respectively, and a p-value < 0.05. Protein ID was matched with values from a previously reported peanut transcriptome database. Gene ontology (GO) annotation proteomes were derived from the UniProt-GO annotation database, and the homologous genes of DEPs referenced by the model plant Arabidopsis database (TAIR, www.arabidopsis.org). DEPs involved in FA biosynthesis were subsequently collected via the gene functional annotation.

### 4.6. Total RNA Extraction and Real-Time PCR

Real-time PCR was carried out according to a previously described method [42]. Briefly, total RNA was extracted from 100 mg of seed from both peanut cultivars using Trizol Reagent (Invitrogen, Beijing, China). The concentration and integrity of purified RNA was assessed using a UV-visible spectrophotometer, DNA NanoDrop (Thermo Fisher, Waltham, MA, USA). RNase-free DNaseI (Fermentas, Waltham, MA, USA) was used to remove genomic DNA contaminants, and 1 µg of total RNA was reversely transcribed into cDNA using a PrimeScript RT reagent Kit (Takara, Dalian, China) in accordance with the manufacturer’s instructions. The PCR reaction was conducted in a 20-µL reaction system using SYBR Premix ExTaq™ (TaKaRa, Dalian, China) on an ABI StepOne Plus system. The relative expressions of target genes were calculated with the 2^−ΔΔ*C*T^ method and shown as fold changes relative to the normal-oleic variety, Kainong70. *Ah18S* (forward primer: 5′-ATTCCTAGTAAGCGCGAGTCATCAG-3′, reverse primer: 5′-CAATGATCCTTCCGCAGGTTCAC-3′) was selected as the housekeeping gene, and the mean values for the CT (cycle time) of *Ah18S* were 20.83 and 20.32 in Kainong70 and Kainong176, respectively (Table S4). The primer sequences used in this experiment are listed in Table S5. Each measurement was carried out in triplicate with three biological replicates, and data are expressed as means ± SE.

## Figures and Tables

**Table 1:** Identification of DEPs involved in the FA pathway at each stage of seed development in the low-oleate variety, Kainong70.

ID	Log^2^ Fold Change	Uniprot	Homologous Gene in *Arabidopsis*	Biological Process
kainong70-2 vs. Kainong70-1				
Araip.10018346.1	2.67	P52410	*KAS1*	fatty acid biosynthetic process
Araip.10018212.1	2.54	Q9LX13	*At5g10160*	fatty acid biosynthetic process
Araip.10000461.1	2.13	P49243	*At1g62640*	fatty acid biosynthetic process
Araip.10008089.1	2.12	Q9C9P4	*KAS2*	unsaturated fatty acid metabolic process
Araip.10012310.1	1.97	Q9SLA8	*MOD1*	fatty acid biosynthetic process
Araip.10010555.1	1.83	Q9ZRA2	*HGO*	lipid oxidation
Araip.10003244.1	1.8	Q9SJH7	*CSY3*	lipid oxidation
Araip.10039800.1	1.8	Q8RU07	*EMB3147*	fatty acid biosynthetic process
Araip.10007633.1	1.79	Q06327	*LOX1*	fatty acid biosynthetic process
Araip.10037915.1	1.68	Q9SQI8	*LTA2*	lipid biosynthetic process
Araip.10038396.1	1.66	Q9FLW9	*PKP2*	lipid biosynthetic process
Araip.10036992.1	1.63	Q9CAP8	*LACS9*	fatty acid metabolic process
Araip.10020945.1	1.61	Q9SW21	*ACP4*	fatty acid biosynthetic process
Araip.10019224.1	1.47	O82399	*PMDH1*	lipid oxidation
Araip.10019122.1	1.46	F4JML5	*At4g16800*	lipid oxidation
Araip.10033415.1	1.44	Q9ZP05	*PMDH2*	regulation of lipid catabolic process
Araip.10019397.1	1.27	Q9LD43	*CAC3*	fatty acid biosynthetic process
Araip.10008554.1	1.27	Q9M8L4	*GLPK*	lipid oxidation
Araip.10034912.1	1.27	Q9FLH8	*At5g51830*	lipid biosynthetic process
Araip.10032969.1	1.23	Q9ZVQ3	*GSTZ1*	lipid oxidation
Araip.10039882.1	1.17	Q8L9C4	*KCR1*	fatty acid biosynthetic process
Araip.10034519.1	1.08	O04983	*CAC2*	fatty acid derivative biosynthetic process
Araip.10005278.1	1.03	O04420	*At2g26230*	lipid oxidation
Araip.10021913.1	1.01	Q9FMN0	*SCP2*	lipid oxidation
Araip.10000513.1	−1.04	Q38882	*PLDALPHA1*	fatty acid metabolic process
Araip.10003126.1	−1.04	O22898	*LACS1*	fatty acid metabolic process
Araip.10030492.1	−1.05	Q9T0A0	*LACS4*	fatty acid biosynthetic process
Araip.10004872.1	−1.2	O65202	*ACX1*	lipid oxidation
Araip.10013985.1	−1.29	Q9SCY5	*KINB2*	fatty acid biosynthetic process
Araip.10025239.1	−1.49	Q8RWZ3	*IBR3*	fatty acid metabolic process
Araip.10021376.1	−1.52	Q9FKE9	*At5g45160*	lipid oxidation
Araip.10022760.1	−1.56	Q9LZ31	*CYP77A4*	lipid oxidation
Araip.10000675.1	−1.74	Q8LCU7	*At3g45770*	fatty acid biosynthetic process
kainong70-3 vs. Kainong70-2				
Araip.10019609.1	1.52	Q93W03	*At3g56130*	negative regulation of fatty acid metabolic process
Araip.10037854.1	1.44	Q42134	*PAE2*	lipid oxidation
Araip.10032974.1	−1.11	P42742	*PBF1*	lipid oxidation
Araip.10005498.1	−1.25	Q9SJH7	*CSY3*	lipid oxidation
Araip.10020218.1	−1.34	Q96329	*ACX4*	lipid oxidation
Araip.10008534.1	−1.46	Q94FY7	*VTE1*	fat-soluble vitamin metabolic process
Araip.10030492.1	−1.6	Q9T0A0	*LACS4*	fatty acid biosynthetic process
Araip.10036992.1	−1.64	Q9CAP8	*LACS9*	fatty acid metabolic process
Araip.10014023.1	−2.07	Q9ZPI5	*MFP2*	lipid oxidation
Araip.10038212.1	−2.14	Q8LPS1	*LACS6*	fatty acid biosynthetic process
Kainong70-4 vs. Kainong70-3				
Araip.10021376.1	1.57	Q9FKE9	*At5g45160*	lipid oxidation
Araip.10020945.1	−1.67	Q9SW21	*ACP4*	fatty acid biosynthetic process
Kainong70-5 vs. Kainong70-4				
no identified				
Kainong70-5 vs. Kainong70-4				
Araip.10029908.1	1.87	Q9M9W8	*PLPZETA2*	lipid oxidation
Araip.10020945.1	1.79	Q9SW21	*ACP4*	fatty acid biosynthetic process
Araip.10010590.1	1.45	Q9LDF5	*At3g15290*	fatty acid metabolic process
Araip.10020381.1	1.08	O22832	*FAB2*	fatty acid biosynthetic process

**Table 2:** Identification of DEPs involved in the FA pathway at each stage of seed development in the high-oleate variety, Kainong176.

ID	Log^2^ Fold Change	Uniprot	Homologous Gene in *Arabidopsis*	Biological Process
Kainong176-2 vs. Kainong176-1				
Araip.10018212.1	2.52	Q9LX13	*At5g10160*	fatty acid biosynthetic process
Araip.10018346.1	2.49	P52410	*KAS1*	fatty acid biosynthetic process
Araip.10000461.1	2.4	P49243	*At1g62640*	fatty acid biosynthetic process
Araip.10012310.1	2.24	Q9SLA8	*MOD1*	fatty acid biosynthetic process
Araip.10019224.1	2.11	O82399	*PMDH1*	lipid oxidation
Araip.10003244.1	1.86	Q9SJH7	*CSY3*	lipid oxidation
Araip.10039800.1	1.86	Q8RU07	*EMB3147*	fatty acid biosynthetic process
Araip.10038396.1	1.85	Q9FLW9	*PKP2*	lipid biosynthetic process
Araip.10038248.1	1.85	Q9LUJ7	*PAP85*	lipid storage
Araip.10007633.1	1.81	Q06327	*LOX1*	fatty acid biosynthetic process
Araip.10033415.1	1.78	Q9ZP05	*PMDH2*	regulation of fatty acid oxidation
Araip.10008554.1	1.75	Q9M8L4	*GLPK*	lipid oxidation
Araip.10010555.1	1.74	Q9ZRA2	*HGO*	lipid oxidation
Araip.10008089.1	1.71	Q9C9P4	*KAS2*	lipid biosynthetic process
Araip.10039818.1	1.71	P33207	*At1g24360*	fatty acid biosynthetic process
Araip.10037915.1	1.67	Q9SQI8	*LTA2*	lipid biosynthetic process
Araip.10019122.1	1.64	F4JML5	*At4g16800*	fatty acid metabolic process
Araip.10020945.1	1.62	Q9SW21	*ACP4*	fatty acid biosynthetic process
Araip.10014644.1	1.62	Q9SS98	*At3g01570*	lipid storage
Araip.10014038.1	1.34	Q9M7Z1	*BCE2*	fatty acid biosynthetic process
Araip.10039882.1	1.33	Q8L9C4	*KCR1*	fatty acid biosynthetic process
Araip.10004625.1	1.26	Q9FVS9	*CYP96A15*	fatty acid derivative metabolic process
Araip.10034912.1	1.25	Q9FLH8	*At5g51830*	lipid metabolic process
Araip.10032969.1	1.23	Q9ZVQ3	*GSTZ1*	lipid oxidation
Araip.10034519.1	1.13	O04983	*CAC2*	lipid biosynthetic process
Araip.10000107.1	1.02	P56765	*accD*	lipid biosynthetic process
Araip.10036992.1	1	Q9CAP8	*LACS9*	fatty acid metabolic process
Araip.10004872.1	−1.04	O65202	*ACX1*	lipid oxidation
Araip.10003126.1	−1.11	O22898	*LACS1*	fatty acid metabolic process
Araip.10034349.1	−1.25	Q9ZPI6	*AIM1*	lipid oxidation
Araip.10003587.1	−1.3	Q9FFE6	*AAE5*	fatty acid metabolic process
Araip.10023165.1	−1.42	P0CZ23	*ACX3*	lipid oxidation
Araip.10017616.1	−1.49	O82265	*SCC3*	lipid oxidation
Araip.10000675.1	−1.55	Q8LCU7	*At3g45770*	fatty acid biosynthetic process
Araip.10025239.1	−1.71	Q8RWZ3	*IBR3*	fatty acid metabolic process
Kainong176-3 vs. Kainong176-2				
Araip.10027909.1	2.74	Q93Y35	*RPN7*	lipid oxidation
Araip.10018302.1	2.3	Q9SGW3	*RPN12A*	lipid oxidation
Araip.10021150.1	2.2	Q06588	*ACO4*	lipid oxidation
Araip.10020381.1	1.97	O22832	*FAB2*	fatty acid biosynthetic process
Araip.10019397.1	1.96	Q9LD43	*CAC3*	fatty acid biosynthetic process
Araip.10039882.1	1.95	Q8L9C4	*KCR1*	fatty acid biosynthetic process
Araip.10003887.1	1.91	O80992	*PYL2*	fatty acid metabolic process
Araip.10032969.1	1.89	Q9ZVQ3	*GSTZ1*	lipid oxidation
Araip.10031916.1	1.79	Q9LLC1	*BCCP2*	fatty acid biosynthetic process
Araip.10031864.1	1.69	Q38997	*KIN10*	lipid biosynthetic process
Araip.10004243.1	1.48	Q9FLW9	*PKP2*	lipid biosynthetic process
Araip.10018529.1	1.46	Q9ZRW8	*GSTU19*	lipid oxidation
Araip.10019122.1	1.44	F4JML5	*At4g16800*	fatty acid beta-oxidation
Araip.10035390.1	1.42	Q9SEI4	*RPT3*	lipid oxidation
Araip.10000267.1	1.4	Q9M2U2	*ECR*	fatty acid biosynthetic process
Araip.10037915.1	1.38	Q9SQI8	*LTA2*	lipid biosynthetic process
Araip.10020218.1	1.33	Q96329	*ACX4*	lipid oxidation
Araip.10006248.1	1.32	Q9S9W2	*SDRA*	fatty acid metabolic process
Araip.10034583.1	1.29	Q9SS98	*At3g01570*	lipid storage
Araip.10034519.1	1.18	O04983	*CAC2*	lipid biosynthetic process
Araip.10007455.1	1.16	Q8GRT9	*At3g15690*	cellular lipid metabolic process
Araip.10028882.1	1.15	Q96242	*CYP74A*	fatty acid derivative metabolic process
Araip.10039818.1	1.09	P33207	*At1g24360*	fatty acid biosynthetic process
Araip.10007632.1	1.01	Q06327	*LOX1*	fatty acid biosynthetic process
Araip.10036036.1	−1.05	Q9C5U1	*AHK3*	response to lipid
Araip.10020945.1	−1.11	Q9SW21	*ACP4*	fatty acid biosynthetic process
Araip.10018212.1	−1.24	Q9LX13	*At5g10160*	fatty acid biosynthetic process
Araip.10024168.1	−1.25	Q8LBB2	*KING1*	fatty acid biosynthetic process
Araip.10014023.1	−1.29	Q9ZPI5	*MFP2*	lipid oxidation
Araip.10027878.1	−1.39	Q9LST0	*At5g60160*	lipid oxidation
Araip.10018115.1	−1.49	Q8VXZ7	*AGAL3*	lipid oxidation
Araip.10039592.1	−1.52	Q9SIE3	*At2g22230*	lipid biosynthetic process
Araip.10034411.1	−1.61	Q7DLS1	*PBB2*	lipid oxidation
Araip.10013255.1	−1.63	O65201	*ACX2*	lipid oxidation
Araip.10023663.1	−1.69	Q9LT08	*RPN11*	lipid oxidation
Araip.10010590.1	−1.77	Q9LDF5	*At3g15290*	fatty acid metabolic process
Araip.10030656.1	−1.79	Q8GYB8	*OPR2*	fatty acid biosynthetic process
Araip.10024291.1	−1.85	B9DGD6	*ACS*	fatty acid biosynthetic process
Araip.10029015.1	−1.94	Q944G9	*FBA2*	lipid biosynthetic process
Araip.10013985.1	−2.29	Q9SCY5	*KINB2*	fatty acid biosynthetic process
Araip.10010555.1	−2.33	Q9ZRA2	*HGO*	lipid oxidation
Araip.10001867.1	−2.51	Q8S4Y1	*AAT1*	cellular lipid catabolic process
Kainong176-4 vs. Kainong176-3				
Araip.10028103.1	2.39	Q9FKE9	*At5g45160*	lipid oxidation
Araip.10023165.1	1.5	P0CZ23	*ACX3*	lipid oxidation
Araip.10037915.1	−1.1	Q9SQI8	*LTA2*	unsaturated fatty acid biosynthetic process
Araip.10030536.1	−1.13	F4HUK6	*AAE1*	fatty acid metabolic process
Araip.10003887.1	−1.37	O80992	*PYL2*	fatty acid metabolic process
Araip.10032969.1	−1.57	Q9ZVQ3	*GSTZ1*	lipid oxidation
Araip.10019122.1	−1.63	F4JML5	*At4g16800*	fatty acid metabolic process
Araip.10039818.1	−2.14	P33207	*At1g24360*	fatty acid metabolic process
Kainong176-5 vs. Kainong176-4				
no identified				
Kainong176-6 vs. Kainong176-5				
Araip.10015142.1	−2.02	Q9M8L4	*GLPK*	lipid oxidation

**Table 3:** Identification of DEPs involved in FA pathways at each stage of seed development in Kainong176 and Kainong70.

ID	Log^2^ Fold Change	Uniprot	Homologous Gene in *Arabidopsis*	Biological Process	Seed Development Stage
Araip.10001989.1	−1.5	Q9FJ62	*GDPDL4*	lipid metabolic process	1
Araip.10013985.1	1.34	Q9SCY5	*KINB2*	fatty acid biosynthetic	2
Araip.10032497.1	−1.03	Q9C826	*ABA2*	lipid metabolic process	2
Araip.10039818.1	2.03	P33207	*At1g24360*	fatty acid biosynthetic	3
Araip.10020381.1	1.82	O22832	*FAB2*	fatty acid biosynthetic	3
Araip.10019122.1	1.62	F4JML5	*At4g16800*	fatty acid metabolic process	3
Araip.10012320.1	1.54	Q9SYT0	*ANN1*	fatty acid metabolic process	3
Araip.10037915.1	1.37	Q9SQI8	*LTA2*	fatty acid metabolic process	3
Araip.10021150.1	2.06	Q06588	*ACO4*	lipid metabolic process	3
Araip.10007105.1	1.42	O64688	*E1-BETA-2*	lipid metabolic process	3
Araip.10033916.1	1.23	P0DKC6	*HSD1*	lipid metabolic process	3
Araip.10037384.1	1.11	Q9C8P0	*EMB3003*	carbohydrate metabolic process	3
Araip.10026147.1	−1.34	F4JQJ7	*At4g36945*	lipid biosynthetic	3
Araip.10023165.1	−1.64	P0CZ23	*ACX3*	lipid oxidation	3
Araip.10006287.1	−1.64	O65390	*APA1*	lipid metabolic process	3
Araip.10033474.1	−1.72	F4J7G5	*At3g11780*	lipid metabolic process	3
Araip.10015601.1	−2.51	Q9M153	*At4g01130*	GDSL-like Lipase	3
Araip.10040083.1	1.31	Q38862	*IPS2*	lipid Inositol-3-phosphate synthase	4
Araip.10017616.1	1.03	O82265	*SCC3*	lipid oxidation	4
Araip.10003529.1	−2	Q9ZVI9	*PECT1*	phospholipid metabolic process	4
Araip.10026823.1	−2.15	Q9LDB4	*LTP6*	non-specific lipid-transfer protein 10-related	4
Araip.10040083.1	1.66	Q38862	*IPS2*	lipid Inositol-3-phosphate synthase	5
Araip.10010555.1	2.07	Q9ZRA2	*HGO*	lipid oxidation	6
Araip.10024204.1	1.53	Q8L7U0	*At3g03330*	lipid oxidation	6
Araip.10006678.1	1.35	Q9FMA3	*PEX5*	lipid oxidation	6
Araip.10033474.1	1.97	F4J7G5	*At3g11780*	lipid metabolic process	6
Araip.10020381.1	−1.18	O22832	*FAB2*	fatty acid biosynthetic	6
Araip.10005278.1	−1.51	O04420	*At2g26230*	lipid oxidation	6

## Supplementary Materials

The following are available online at http://www.mdpi.com/1422-0067/19/4/1235/s1.

## References

[1] Akhtar S., Khalid N., Ahmed I., Shahzad A., Suleria H.A. (2014). Physicochemical characteristics, functional properties, and nutritional benefits of peanut oil: A review. Crit. Rev. Food Sci. Nutr..

[2] Zhao C., Qiu J., Agarwal G., Wang J., Ren X., Xia H., Guo B., Ma C., Wan S., Bertioli D.J. (2017). Genome-Wide Discovery of Microsatellite Markers from Diploid Progenitor Species, *Arachis duranensis* and *A. ipaensis*, and Their Application in Cultivated Peanut (*A. hypogaea*). Front. Plant Sci..

[3] Zhang L., Yang X., Tian L., Chen L., Yu W. (2016). Identification of peanut (*Arachis hypogaea*) chromosomes using a fluorescence in situ hybridization system reveals multiple hybridization events during tetraploid peanut formation. New Phytol..

[4] Chen X., Li H., Pandey M.K., Yang Q., Wang X., Garg V., Li H., Chi X., Doddamani D., Hong Y. (2016). Draft genome of the peanut A-genome progenitor (*Arachis duranensis*) provides insights into geocarpy, oil biosynthesis, and allergens. Proc. Natl. Acad. Sci. USA.

[5] Bertioli D.J., Cannon S.B., Froenicke L., Huang G., Farmer A.D., Cannon E.K., Liu X., Gao D., Clevenger J., Dash S. (2016). The genome sequences of *Arachis duranensis* and *Arachis ipaensis*, the diploid ancestors of cultivated peanut. Nat. Genet..

[6] Cernay C., Pelzer E., Makowski D. (2016). A global experimental dataset for assessing grain legume production. Sci. Data.

[7] Vassiliou E.K., Gonzalez A., Garcia C., Tadros J.H., Chakraborty G., Toney J.H. (2009). Oleic acid and peanut oil high in oleic acid reverse the inhibitory effect of insulin production of the inflammatory cytokine TNF-α both in vitro and in vivo systems. Lipids Health Dis..

[8] Okuley J., Lightner J., Feldmann K., Yadav N., Lark E., Browse J. (1994). *Arabidopsis FAD2* gene encodes the enzyme that is essential for polyunsaturated lipid synthesis. Plant Cell.

[9] Patel M., Jung S., Moore K., Powell G., Ainsworth C., Abbott A. (2004). High-oleate peanut mutants result from a MITE insertion into the *FAD2* gene. Theor. Appl. Genet..

[10] Wang Y., Ma X., Zhang X., He X., Li H., Cui D., Yin D. (2016). ITRAQ-Based Proteomic Analysis of the Metabolic Mechanisms behind Lipid Accumulation and Degradation during Peanut Seed Development and Postgermination. J. Proteome Res..

[11] Li H.F., Zhu F.H., Li H.Y., Zhu W., Chen X.P., Hong Y.B., Liu H.Y., Wu H., Liang X.Q. (2013). Proteomic identification of gravitropic response genes in peanut gynophores. J. Proteom..

[12] Zhao C., Zhao S., Hou L., Xia H., Wang J., Li C., Li A., Li T., Zhang X., Wang X. (2015). Proteomics analysis reveals differentially activated pathways that operate in peanut gynophores at different developmental stages. BMC Plant Biol..

[13] Yang W., Dong R., Liu L., Hu Z., Li J., Wang Y., Ding X., Chu Z. (2016). A novel mutant allele of *SSI2* confers a better balance between disease resistance and plant growth inhibition on *Arabidopsis thaliana*. BMC Plant Biol..

[14] Mekhedov S., de Ilarduya O.M., Ohlrogge J. (2000). Toward a functional catalog of the plant genome. A survey of genes for lipid biosynthesis. Plant Physiol..

[15] Carlsson A.S., LaBrie S.T., Kinney A.J., von Wettstein-Knowles P., Browse J. (2002). A *KAS2* cDNA complements the phenotypes of the *Arabidopsis fab1* mutant that differs in a single residue bordering the substrate binding pocket. Plant J..

[16] Vellosillo T., Martinez M., Lopez M.A., Vicente J., Cascón T., Dolan L., Hamberg M., Castresana C. (2007). Oxylipins produced by the 9-lipoxygenase pathway in *Arabidopsis* regulate lateral root development and defense responses through a specific signaling cascade. Plant Cell.

[17] Shockey J.M., Fulda M.S., Browse J.A. (2002). *Arabidopsis* contains nine long-chain acyl-coenzyme a synthetase genes that participate in fatty acid and glycerolipid metabolism. Plant Physiol..

[18] Beaudoin F., Gable K., Sayanova O., Dunn T., Napier J.A. (2002). A Saccharomyces cerevisiae gene required for heterologous fatty acid elongase activity encodes a microsomal β-keto-reductase. J. Biol. Chem..

[19] Branen J.K., Shintani D.K., Engeseth N.J. (2003). Expression of antisense acyl carrier protein-4 reduces lipid content in *Arabidopsis* leaf tissue. Plant Physiol..

[20] Ke J., Wen T.N., Nikolau B.J., Wurtele E.S. (2000). Coordinate regulation of the nuclear and plastidic genes coding for the subunits of the heteromeric acetyl-coenzyme A carboxylase. Plant Physiol..

[21] Froman B.E., Edwards P.C., Bursch A.G., Dehesh K. (2000). ACX3, a novel medium-chain acyl-coenzyme A oxidase from *Arabidopsis*. Plant Physiol..

[22] De Boer G.J., Testerink C., Pielage G., Nijkamp H.J., Stuitje A.R. (1999). Sequences surrounding the transcription initiation site of the *Arabidopsis* enoyl-acyl carrier protein reductase gene control seed expression in transgenic tobacco. Plant Mol. Biol..

[23] Zhao L., Katavic V., Li F., Haughn G.W., Kunst L. (2010). Insertional mutant analysis reveals that long-chain acyl-CoA synthetase 1 (*LACS1*), but not *LACS8*, functionally overlaps with *LACS9* in *Arabidopsis* seed oil biosynthesis. Plant J..

[24] Jessen D., Roth C., Wiermer M., Fulda M. (2015). Two activities of long-chain acyl-coenzyme A synthetase are involved in lipid trafficking between the endoplasmic reticulum and the plastid in *Arabidopsis*. Plant Physiol..

[25] Kachroo P., Shanklin J., Shah J., Whittle E.J., Klessig D.F. (2001). A fatty acid desaturase modulates the activation of defense signaling pathways in plants. Proc. Natl. Acad. Sci. USA.

[26] Dehesh K., Tai H., Edwards P., Byrne J., Jaworski J.G. (2001). Overexpression of 3-ketoacyl-acyl-carrier protein synthase IIIs in plants reduces the rate of lipid synthesis. Plant Physiol..

[27] Wu G.Z., Xue H.W. (2010). *Arabidopsis* beta-ketoacyl-[acyl carrier protein] synthase is crucial for fatty acid synthesis and plays a role in chloroplast division and embryo development. Plant Cell.

[28] Beld J., Lee D.J., Burkart M.D. (2015). Fatty acid biosynthesis revisited: Structure elucidation and metabolic engineering. Mol. Biosyst..

[29] Kachroo A., Shanklin J., Whittle E., Lapchyk L., Hildebrand D., Kachroo P. (2007). The *Arabidopsis* stearoyl-acyl carrier protein-desaturase family and the contribution of leaf isoforms to oleic acid synthesis. Plant Mol. Biol..

[30] Block M.A., Jouhet J. (2015). Lipid trafficking at endoplasmic reticulum-chloroplast membrane contact sites. Curr. Opin. Cell Biol..

[31] ALJohani A.M., Syed D.N., Ntambi J.M. (2017). Insights into Stearoyl-CoA Desaturase-1 Regulation of Systemic Metabolism. Trends Endocrinol. Metab..

[32] Chi X., Yang Q., Pan L., Chen M., He Y., Yang Z., Yu S. (2011). Isolation and characterization of fatty acid desaturase genes from peanut (*Arachis hypogaea* L.). Plant Cell Rep..

[33] Dar A.A., Choudhury A.R., Kancharla P.K., Arumugam N. (2017). The *FAD2* Gene in Plants: Occurrence, Regulation, and Role. Front. Plant Sci..

[34] Bowen K.J., Kris-Etherton P.M., Shearer G.C., West S.G., Reddivari L., Jones P. (2017). Oleic acid-derived oleoylethanolamide: A nutritional science perspective. Prog. Lipid Res..

[35] Lung S.C., Chye M.L. (2016). Acyl-CoA-Binding Proteins (ACBPs) in Plant Development. Subcell. Biochem..

[36] Mooney B.P., Miernyk J.A., Randall D.D. (2002). The complex fate of alpha-ketoacids. Ann. Rev. Plant Biol..

[37] LeClere S., Rampey R.A., Bartel B. (2004). *IAR4*, a gene required for auxin conjugate sensitivity in *Arabidopsis*, encodes a pyruvate dehydrogenase E1α homolog. Plant Physiol..

[38] Taylor N.L., Heazlewood J.L., Day D.A., Millar A.H. (2004). Lipoic acid-dependent oxidative catabolism of α-keto acids in mitochondria provides evidence for branched-chain amino acid catabolism in Arabidopsis. Plant Physiol..

[39] Gupta K., Kayam G., Faigenboim-Doron A., Clevenger J., Ozias-Akins P., Hovav R. (2016). Gene expression profiling during seed-filling process in peanut with emphasis on oil biosynthesis networks. Plant Sci..

[40] Wisniewski J.R., Zougman A., Nagaraj N., Mann M. (2009). Universal sample preparation method for proteome analysis. Nat. Methods..

[41] Zheng X., Fan S., Wei H., Tao C., Ma Q., Ma Q., Zhang S., Li H., Pang C., Yu S. (2017). iTRAQ-Based Quantitative Proteomic Analysis Reveals Cold Responsive Proteins Involved in Leaf Senescence in Upland Cotton (*Gossypium hirsutum* L.). Int. J. Mol. Sci..

[42] Liu H., Dong S., Gu F., Liu W., Yang G., Huang M., Xiao W., Liu Y., Guo T., Wang H. (2017). NBS-LRR Protein Pik-H4 Interacts with OsBIHD1 to Balance Rice Blast Resistance and Growth by Coordinating Ethylene-Brassinosteroid Pathway. Front Plant Sci..

