# Supplementary material for: Identification of the Candidate Proteins Related to Oleic Acid Accumulation during Peanut (Arachis hypogaea L.) Seed Development through Comparative Proteome Analysis
Source: Int J Mol Sci. 2018 Apr 18;19(4):1235. doi: 10.3390/ijms19041235 (PMC5979506; doi:10.3390/ijms19041235)
Supplement: Supplementary file 1 [file ijms-19-01235-s001.zip › supplementary files/supplementary file.pdf]

Identification of the Candidate Proteins Related to Oleic Acid Accumulation during Peanut (*Arachis hypogaea L.*) Seed Development through Comparative Proteome Analysis

Author names:

Hao Liu[1#], Haifen Li[1#], Jianzhong Gu[2], Li Deng[2] , Li Ren[2], Yanbin Hong [1], Qing Lu[1], Xiaoping Chen[1*], Xuanqiang Liang[1*]

# For co-first author, these authors contributed equally to this work.

* For corresponding.

Affiliations:

[1] Crops Research Institute, Guangdong Academy of Agricultural Sciences, South China Peanut Sub-Center of National Center of Oilseed Crops Improvement, Guangdong Provincial Key Laboratory of Crop Genetic Improvement, Guangzhou, China.

[2] Peanut Research Institute, Kaifeng Academy of Agricultural Sciences, Kaifeng 475004, China

***Co-corresponding author**:

Xiaoping Chen,

E-mail: chenxiaoping@gdaas.cn

***Corresponding author**:

Xuanqiang Liang,

E-mail: liangxuanqiang@gdaas.cn

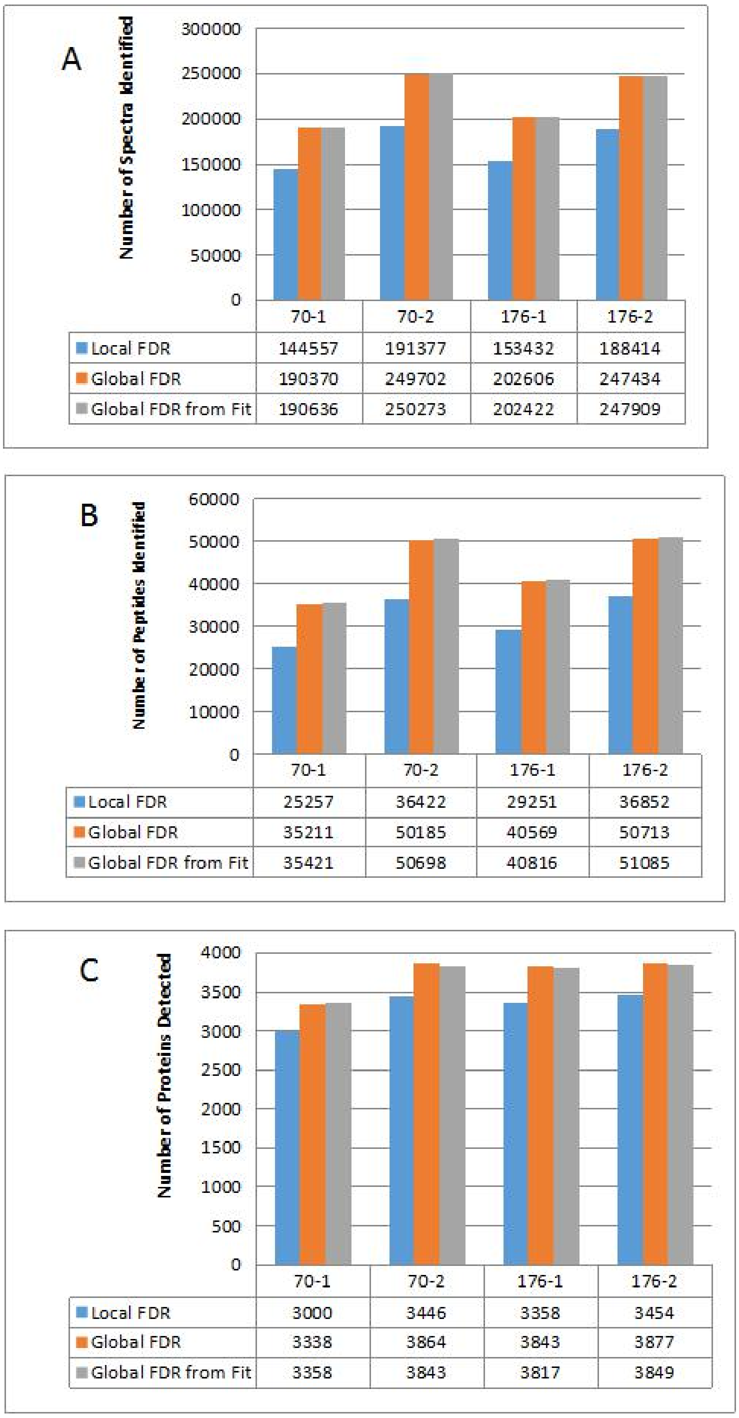

	70-1	70-2	176-1	176-2
Local FDR	144557	191377	153432	188414
Global FDR	190370	249702	202606	247434
Global FDR from Fit	190636	250273	202422	247909

	70-1	70-2	176-1	176-2
Local FDR	25257	36422	29251	36852
Global FDR	35211	50185	40569	50713
Global FDR from Fit	35421	50698	40816	51085

	70-1	70-2	176-1	176-2
Local FDR	3000	3446	3358	3454
Global FDR	3338	3864	3843	3877
Global FDR from Fit	3358	3843	3817	3849

Figure S1. Results of mass spectrometry analysis and protein identification. A-C, Number statistics of spectra, peptides, and proteins in the samples from different biological replicate.

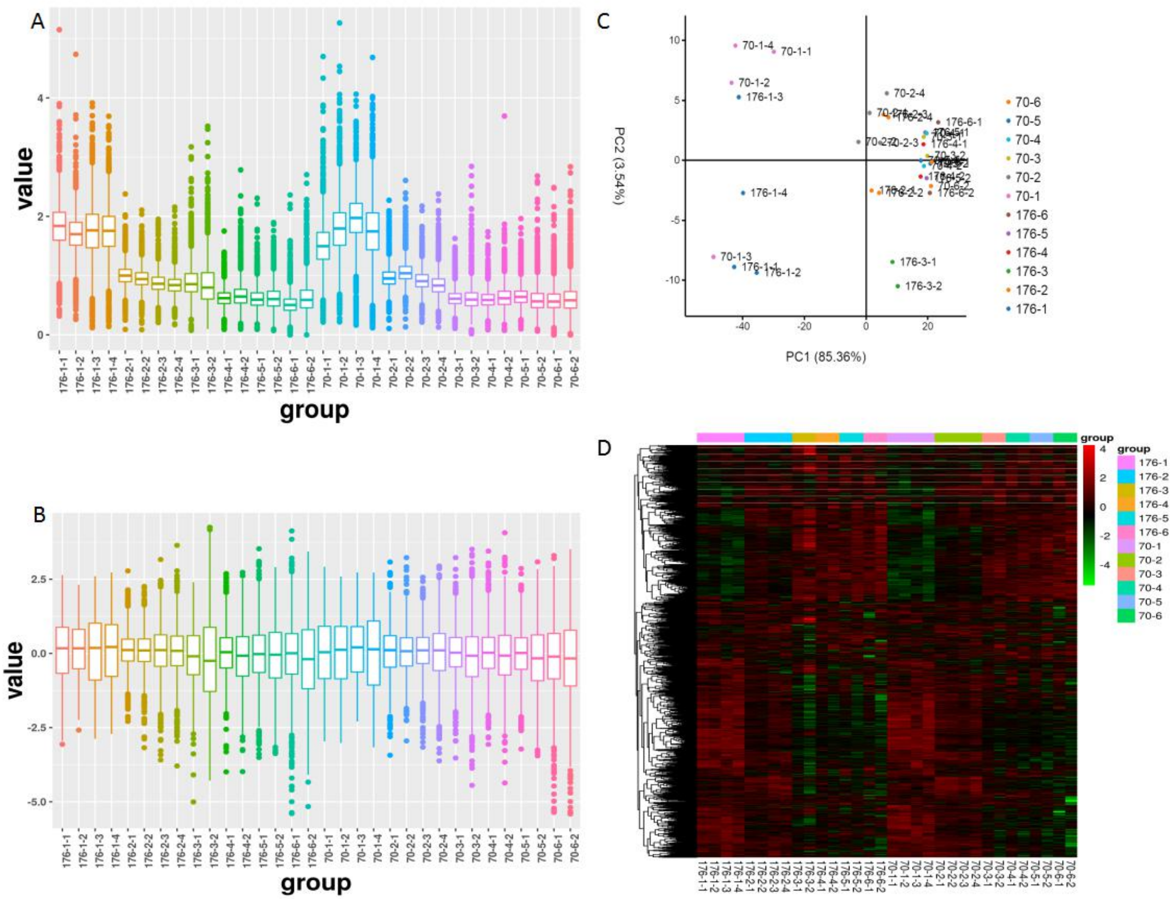

Figure S2. Quality control of experimental data. A-B, normalization treatment of experimental data. C, Principal component analysis (PCA) of different samples from different biological replicate. D, Heatmap represented the level expression of total differentially expressed proteins across the comparable samples Kainong70 and Kainong176.

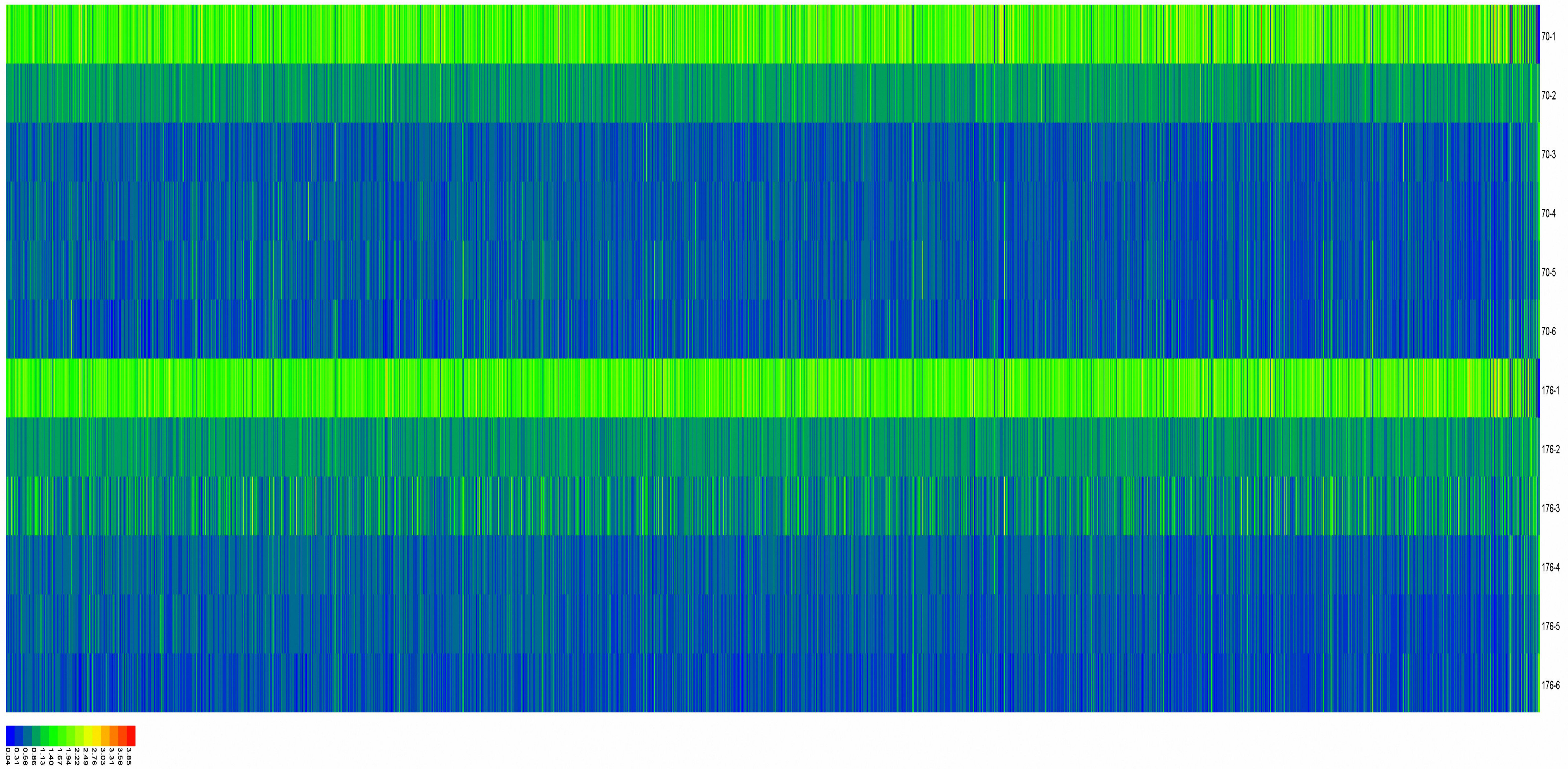

Figure S3. Heatmap displayed the expression levels of identified DEPs in both peanut cultivars. The number (70-1, 70-2,......, 176-6) in the heatmap indicated the samples came from 6 development stages (1-6) in normal-oleate cultivar Kainong70 (70) and high-oleate cultivar Kainong176(176).

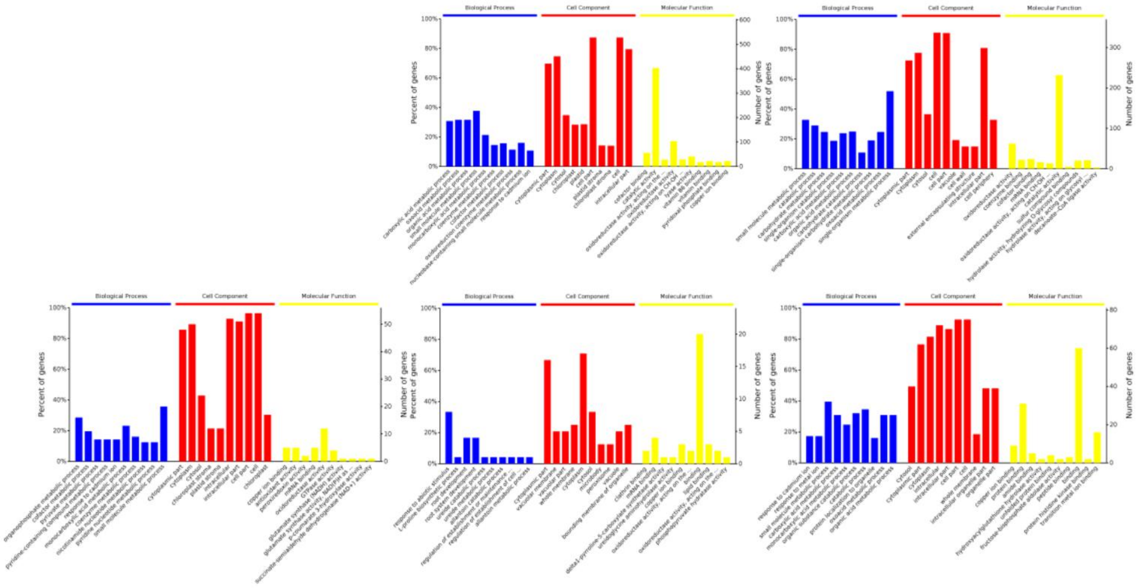

Figure S4. Histogram showing GO classification of DEPs at each stage of seed development in low-oleate variety Kainong70.

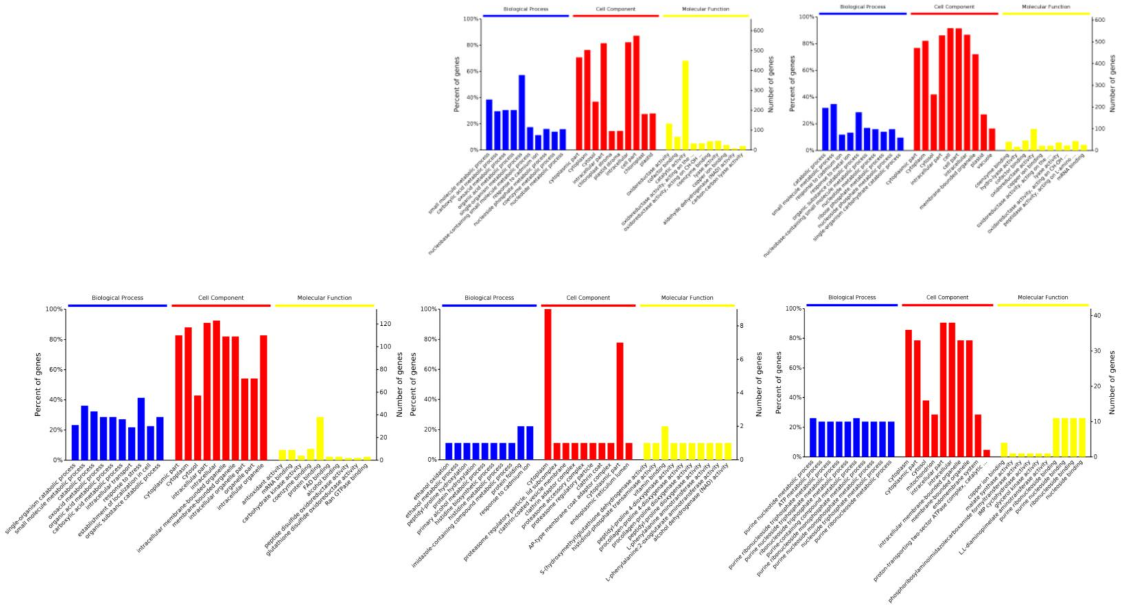

Figure S5. Histogram showing GO classification of DEPs at each stage of seed development in high-oleate variety Kainong176.

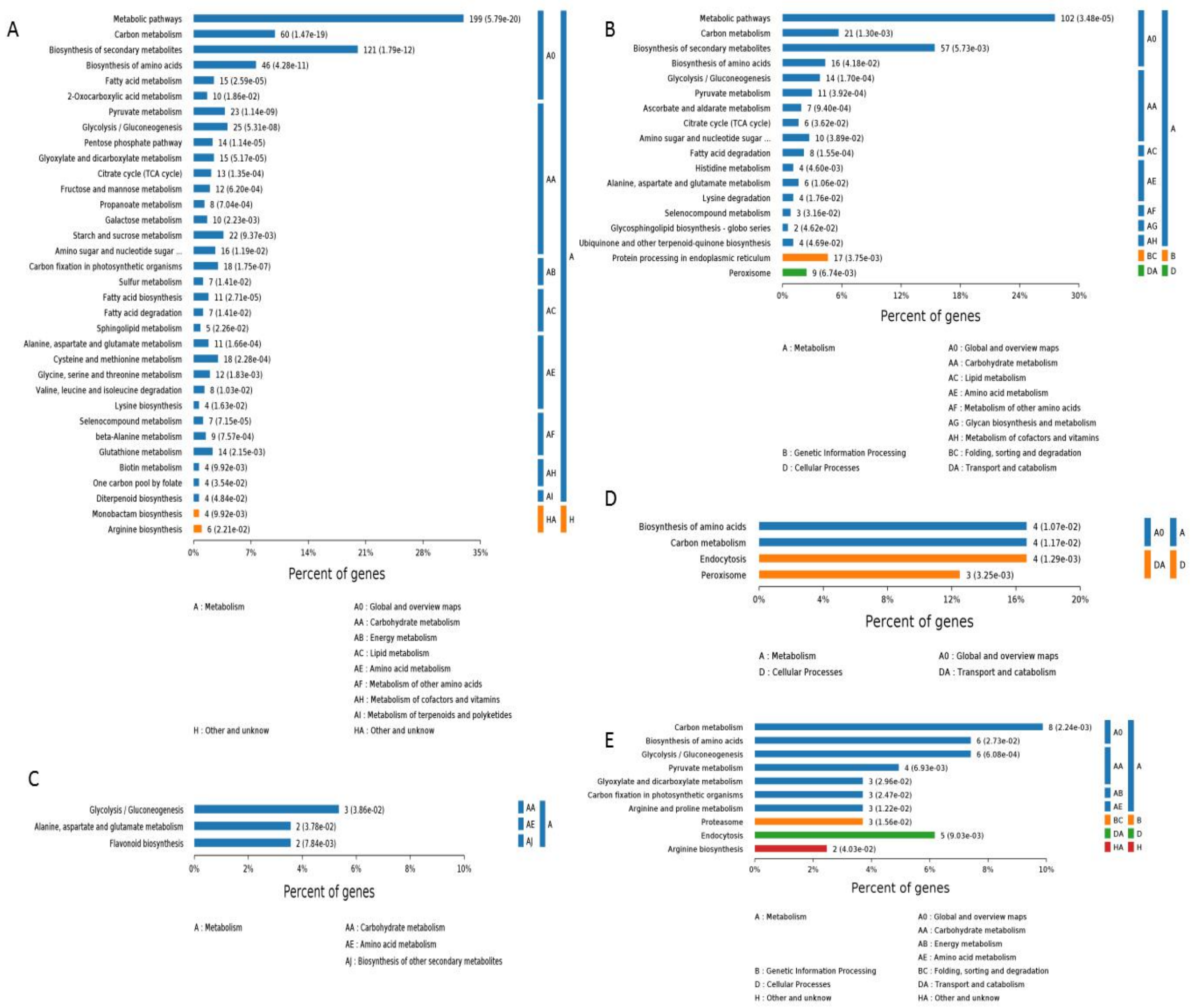

Figure S6. KEGG pathway enrichment of DEPs during the seed development in low-oleate variety Kainong70. From A to E represented the 70-2VS70-1, 70-3VS70-2, 70-4VS70-3, 70-5VS70-4, and 70-6VS70-5, respectively.

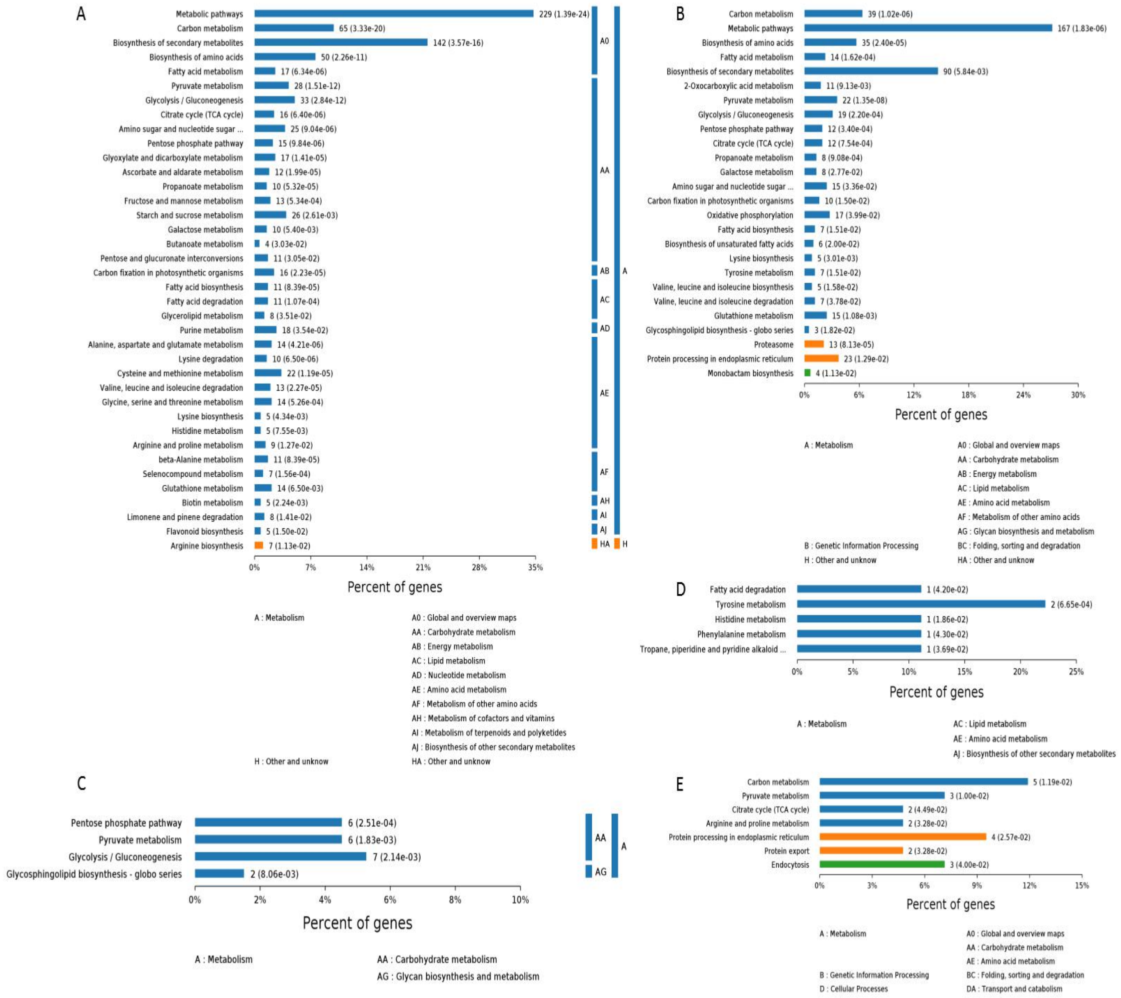

Figure S7. KEGG pathway enrichment of DEPs during the seed development in high-oleate variety Kainong176. From A to E represented the 176-2VS176-1, 176-3VS176-2, 176-4VS176-3, 176-5VS176-4, and 176-6VS176-5, respectively.

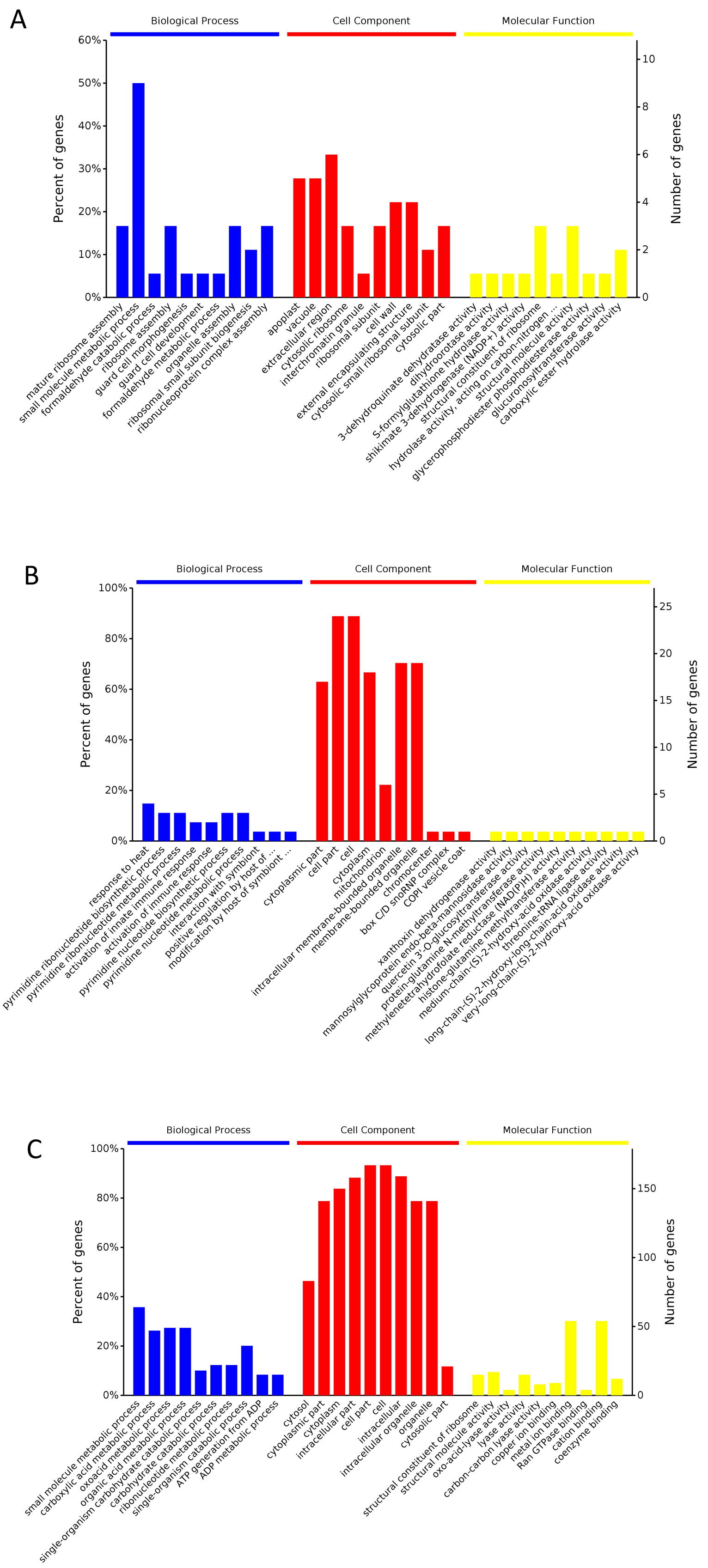
A
Biological Process
Cell Component
Molecular Function
Percent of genes
Number of genes
0%
10%
20%
30%
40%
50%
60%
0
2
4
6
8
10
mature ribosome assembly
small molecule metabolic process
formaldehyde catabolic process
ribosome assembly
guard cell morphogenesis
guard cell development
formaldehyde metabolic process
organelle assembly
ribosomal small subunit biogenesis
ribonucleoprotein complex assembly
apoplast
vacuole
extracellular region
cytosolic ribosome
interchromatin granule
ribosomal subunit
cell wall
external encapsulating structure
cytosolic small ribosomal subunit
cytosolic part
3-dehydroquinate dehydratase activity
dihydroorotase activity
S-formylglutathione hydrolase activity
shikimate 3-dehydrogenase (NADP+) activity
structural constituent of ribosome
hydrolase activity, acting on carbon-nitrogen ...
structural molecule activity
glycerophosphodiester phosphodiesterase activity
glucuronosyltransferase activity
carboxylic ester hydrolase activity
B
Biological Process
Cell Component
Molecular Function
Percent of genes
Number of genes
0%
20%
40%
60%
80%
100%
0
5
10
15
20
25
response to heat
pyrimidine ribonucleotide biosynthetic process
pyrimidine ribonucleotide metabolic process
activation of innate immune response
activation of immune response
pyrimidine nucleotide biosynthetic process
pyrimidine nucleotide metabolic process
interaction with symbiont
positive regulation by host of ...
modification by host of symbiont ...
cytoplasmic part
cell part
cell
cytoplasm
mitochondrion
intracellular membrane-bounded organelle
membrane-bounded organelle
chromocenter
box C/D snoRNP complex
COPI vesicle coat
xanthoxin dehydrogenase activity
mannosylglycoprotein endo-beta-mannosidase activity
quercetin 3-O-glucosyltransferase activity
protein-glutamine N-methyltransferase activity
methylenetetrahydrofolate reductase (NAD(P)H) activity
histone-glutamine methyltransferase activity
medium-chain-(S)-2-hydroxy-acid oxidase activity
threonine-tRNA ligase activity
long-chain-(S)-2-hydroxy-long-chain-acid oxidase activity
very-long-chain-(S)-2-hydroxy-acid oxidase activity
C
Biological Process
Cell Component
Molecular Function
Percent of genes
Number of genes
0%
20%
40%
60%
80%
100%
0
50
100
150
small molecule metabolic process
carboxylic acid metabolic process
oxoacid metabolic process
organic acid metabolic process
carbohydrate catabolic process
single-organism carbohydrate catabolic process
ribonucleotide metabolic process
single-organism catabolic process
ATP generation from ADP
ADP metabolic process
cytosol
cytoplasmic part
cytoplasm
intracellular part
cell part
cell
intracellular
intracellular organelle
organelle
cytosolic part
structural constituent of ribosome
structural molecule activity
oxo-acid-lyase activity
lyase activity
carbon-carbon lyase activity
copper ion binding
metal ion binding
Ran GTPase binding
cation binding
coenzyme binding

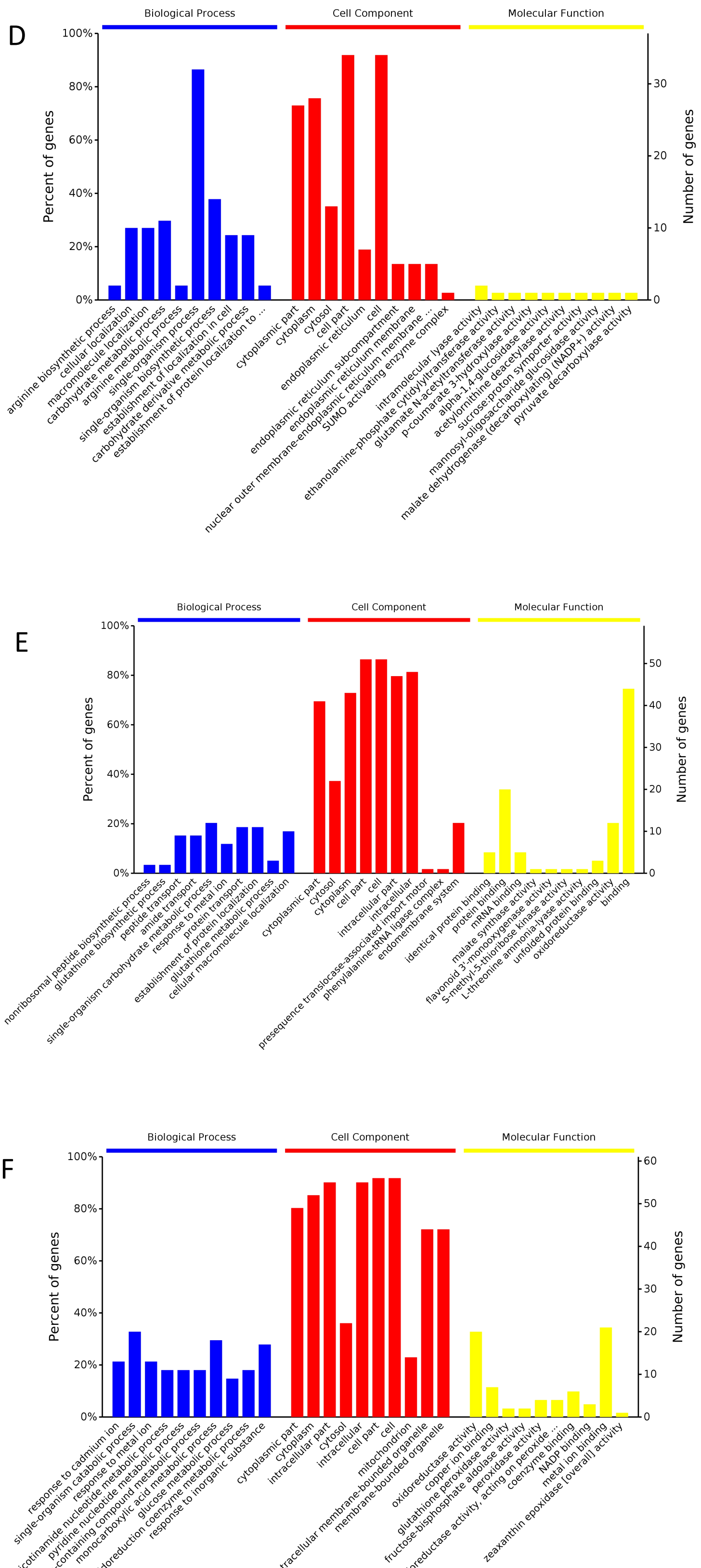

Figure S8 Histogram showing GO classification of DEPs during seed development in Kainong176 vs. Kainong70. A-F, Gene ontology analysis of the differentially expressed

proteins in Kainong176 VS Kainong70 under the different development stage. From A to F represented the 20(stage1), 30(stage2), 40(stage3), 50(stage4), 60(stage5), and 70(stage6) DAF (days after flowering), respectively. Most top 10 terms were displayed in the biological process, cell component, and molecular function process, respectively.

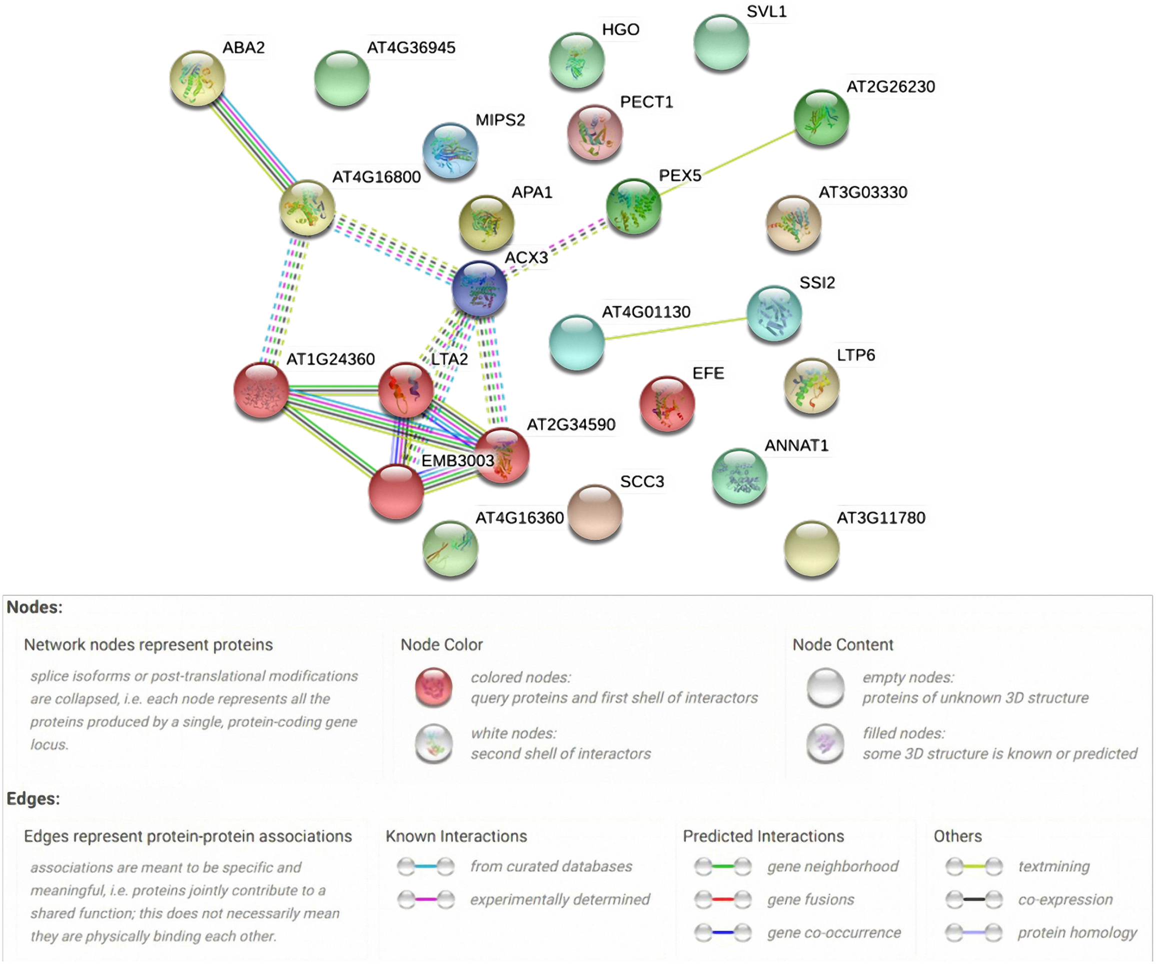

Figure S9. STRING predicted the interacting network of 28 DEPs regarding FA synthesis and metabolism using their homologous genes in *Arabidopsis*. In the STRING network, the nodes represented the proteins; and the results in the nodes represented the predicted 3D structures of proteins. The lines represented the associated relationship between the linked proteins, different line colors indicated evidence of the interaction between the linked proteins. For example, blue and pink solid line indicated the curated database and experimentally determined real protein-protein associated evidence, but the dotted lines represented the putative interaction evidence based on bioinformatics analysis of database, and the network was clustered to a specified MCL inflation parameter.

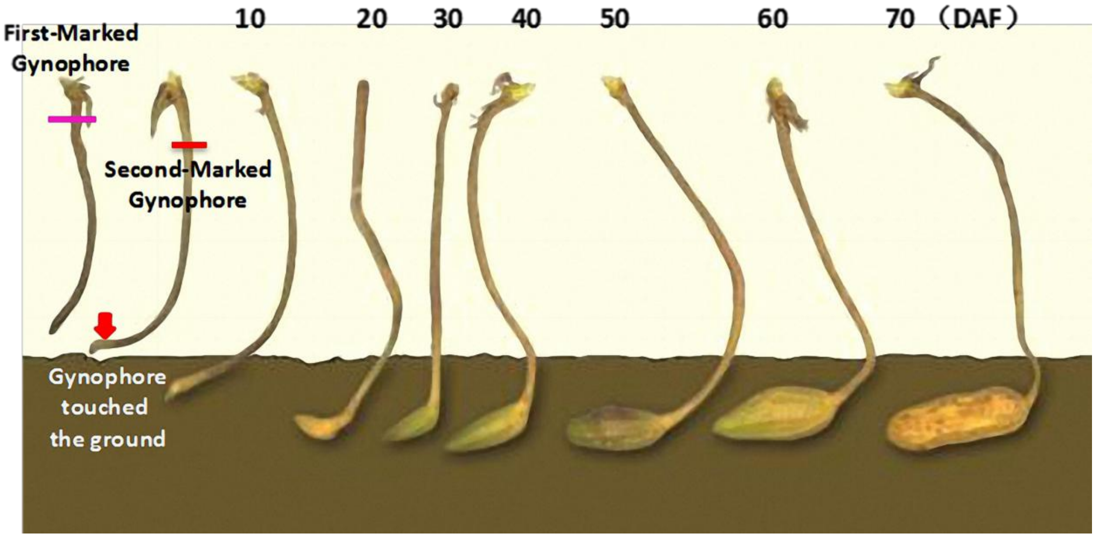

Figure S10. The process of marked gynophore during peanut seed development. We made a cartoon to explain the process of seeds collection in two peanut cultivars, but the picture was downloaded from a website (www.baidu.com) just for explain the question, not for commercial use.

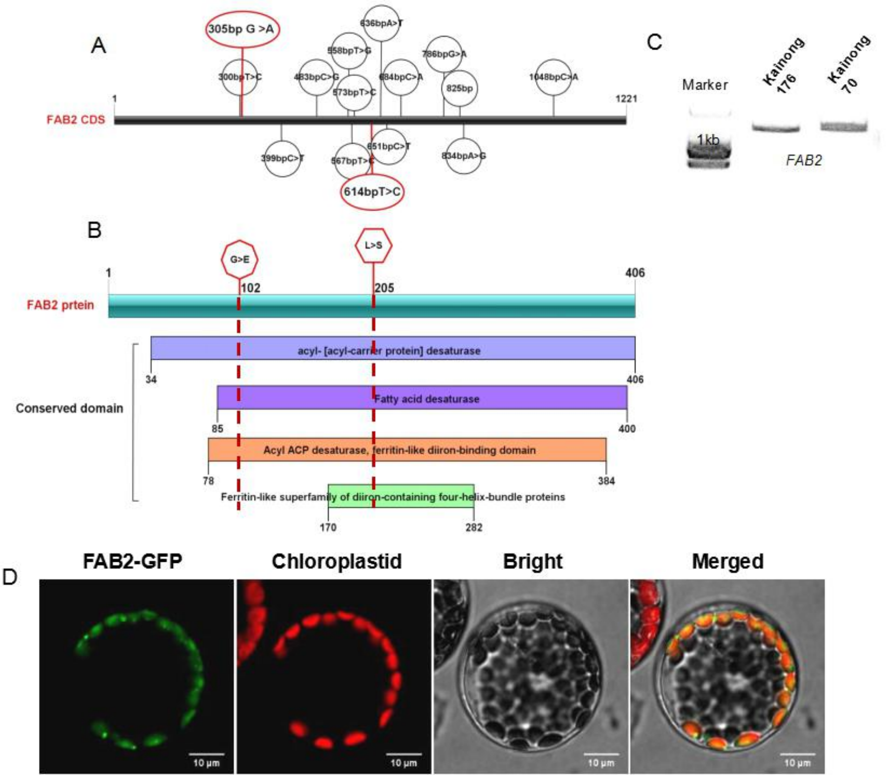

Figure S11. Recent study results of *FAB2*. A, 16 mutant site of bases in high-oleic cultivar Kainong176 compared with Kainong70. B, protein structure of *FAB2* in Kainong176. C, *FAB2* coding sequences were detected by agarose gel electrophoresis in different cultivars. D, subcellular localization of *FAB2-GFP* in protoplast cell of *Arabidopsis*.

Table S5. CT-value of *Ah18S* in the real-time PCR reaction.

	**Cycle Time (CT)**	**Stage 1**	**Stage 2**	**Stage 3**	**Stage 4**	**Stage 5**	**Stage 6**	CT Mean (stage)
CT(Kainong70)	Repeat 1	21.69733429	21.84574127	20.15601921	20.2365818	20.55398369	20.8539772	20.89060624
	Repeat 2	21.29128265	21.69669533	20.08484459	20.65895844	20.20822525	21.11344147	20.84224129
	Repeat 3	21.28997612	21.68653107	20.29577255	21.00416756	19.81446457	20.53738022	20.77138201
	CT Mean (repeat)	21.42619769	21.74298922	20.17887878	20.63323593	20.1922245	20.83493296	20.83474318
CT(Kainong176)	Repeat 1	20.93497086	20.20988655	19.75986481	19.52990913	20.94015503	20.60691452	20.33028348
	Repeat 2	20.11518478	20.18793106	19.44475746	19.96493912	20.76073647	20.71387291	20.19790363
	Repeat 3	20.95371056	20.0320816	19.24794006	19.79506874	21.26062584	21.3200779	20.43491745
	CT Mean (repeat)	20.6679554	20.14329974	19.48418744	19.76330566	20.98717244	20.88028844	20.32103486
